# Imidazole-amino acids. Conformational switch under tautomer and pH change

**DOI:** 10.1007/s00726-022-03201-0

**Published:** 2022-11-02

**Authors:** Monika Staś, Piotr Najgebauer, Dawid Siodłak

**Affiliations:** Faculty of Chemistry, University of Opole, 45-052 Opole, Poland

**Keywords:** Imidazole, Tautomers, Non-standard amino acids, Conformational analysis, Ramachandran map, Density Functional Theory (DFT)

## Abstract

Replacement of the main chain peptide bond by imidazole ring seems to be a promising tool for the peptide-based drug design, due to the specific prototropic tautomeric as well as amphoteric properties. In this study, we present that both tautomer and pH change can cause a conformational switch of the studied residues of alanine (**1–4**) and dehydroalanine (**5–8**) with the C-terminal peptide group replaced by imidazole. The DFT methods are applied and an environment of increasing polarity is simulated. The conformational maps (Ramachandram diagrams) are presented and the stability of possible conformations is discussed. The neutral forms, tautomers *τ* (**1**) and *π* (**2**), adapt the conformations *αRτ* (*φ*, *ψ* = − 75°, − 114°) and C7eq (*φ*, *ψ* = − 75°, 66°), respectively. Their torsion angles *ψ* differ by about 180°, which results in a considerable impact on the peptide chain conformation. The cation form (**3**) adapts both these conformations, whereas the anion analogue (**4)** prefers the conformations *C*5 (*φ*, *ψ* = − 165°, − 178°) and *β*2 (*φ*, *ψ* ~ − 165°, − 3°). Dehydroamino acid analogues, the tautomers *τ* (**5**) and *π* (**6**) as well as the anion form (**8**), have a strong tendency toward the conformations *β*2 (*φ*, *ψ* = − 179°, 0°) and C5 (*φ*, *ψ* = − 180°, 180°). The preferences of the protonated imidazolium form (**7**) depend on the environment. The imidazole ring, acting as a donor or acceptor of the hydrogen bonds created within the studied residues, has a profound effect on the type of conformation.

**Supplementary Information:**

The online version contains supplementary material available at 10.1007/s00726-022-03201-0.

## Introduction

Unusual amino acid residues containing azole rings in the main chain are found in many bacteria’s metabolites. The most common rings are thiazole, oxazole, and oxazoline. Naturally occurring peptides with these structural elements reveal a broad spectrum of biological activity (Siodłak 2015; Bagley et al. 2005; Davyt and Serra 2010; Jin 2009, 2011, 2013, 2016; Just-Baringo et al. 2014; Craveur et al. 2019; Ding et al. 2020). The introduction of the azole structural motif in the peptide main chain in a post-translational modification (de Brevern and Rebehmed 2022) usually constrains its conformational flexibility (Soor et al. 2018; Walker et al. 2021). On the other hand, azole in the side chain can be used as building blocks for the construction of artificial metalloenzymes (Lenartowicz et al. 2021). Azole rings can be also used as trans replacement agents (Kaczmarek et al. 2021; Lenartowicz et al. 2022). Our previous studies show that the amino acid residues containing azole rings in the main chain possess unusual conformational properties (Siodłak et al. 2014b; Staś et al. 2026a, 2016b, 2021). Thiazole and oxazole amino acids tend to adopt the unique semi-extended conformation *β*2 (*φ*, *ψ* ≈ 180°, 0°), especially in weakly polar environments, where this global conformation is stabilized through the formation of the internal N–H···N hydrogen bond, atypical for standard residues. A more intuitive choice for those amino acids would be the extended conformation C5 (*φ*, *ψ* ≈ 180°, 180°), however, the azomethine nitrogen atom (-N =) is a better hydrogen bond acceptor than the sulphur or oxygen atoms, which makes this conformation less preferable. But what if the sulphur or oxygen were substituted by the second nitrogen atom? To answer this question we choose to study the amino acid units with imidazole, which resemble naturally occurring residues in macrocycles (Siodłak 2015; Bagley et al. 2005; Davyt and Serra 2010; Jin 2009, 2011, 2013, 2016; Just-Baringo et al. 2014). In natural peptides or proteins, a residue with imidazole in the main chain has not been found yet. Such a residue could be biosynthesized from 2,3-diaminopropanoic acid (Dap), however, this amino acid residue rarely occurs in microorganisms (Temperini et al. 2020; Xu et al. 2015; Dobrovinskaya et al. 2008). Nevertheless, the imidazole residues were successfully incorporated into the peptide scaffold in the lab synthesis (You and Kelly 2004; Haberhauer and Rominger 2003; Haberhauer et al. 2007; Haberhauer et al. 2005; Loos et al. 2013). Furthermore, an exceptional feature of imidazole is that it can be protonated and deprotonated, which is a key property of a histidine residue in proton transfer pathways (Khorobrykh and Klimov 2005; Hiroshi and Knapp 2005; Fisher et al. 2005). It has been shown that the neutral imidazole ring structure undergoes reorganization upon protonation (Duboué-Dijon et al. 2017). Moreover, the prototrophic tautomerization in its neutral form is another aspect of imidazole to explore. The presence or absence of the NH group results in the different conformational properties and the formation of different hydrogen bonds pattern (Hamissa et al. 2022). The environment or type and position of substituents influence the equilibrium of tautomers and their properties (Kusakiewicz-Dawid et al. 2019; Podolyan et al. 2003; Kapusta et al. 2021; Alkorta and Elguero 2019). Despite the potential utility of imidazole-amino acids their conformational properties were not so far fully recognised.

**Fig. 1:**
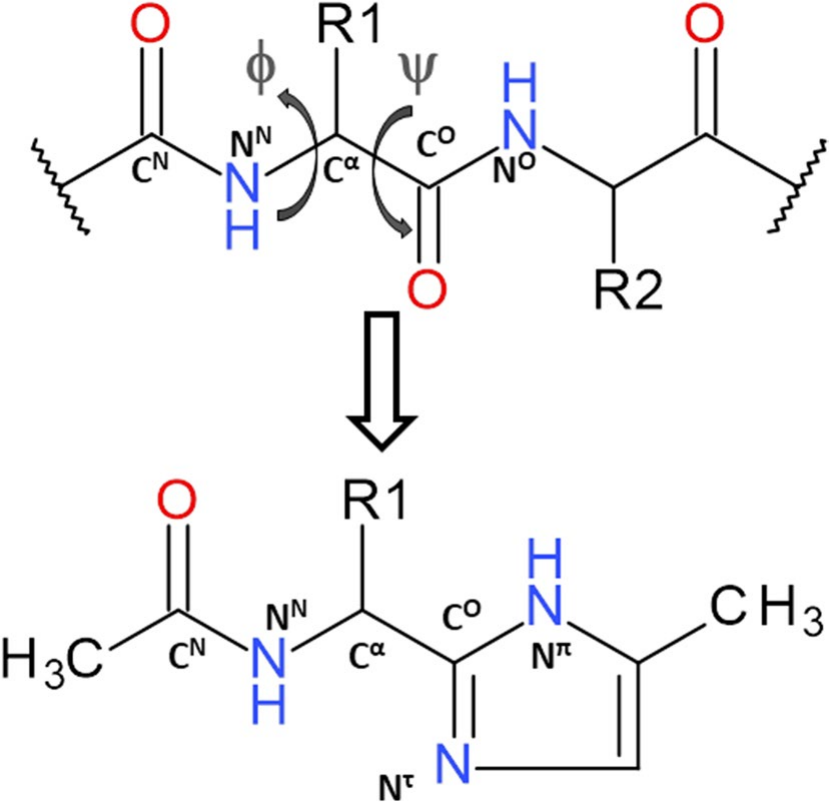
The schematic formula of the studied molecular model containing imidazole-amino acid residue. The torsion angles are based on the following atoms: *φ* (C^*N*^-N^*N*^-C^*α*^-C^*O*^), *ψ* (N^*N*^-C^*α*^-C^*O*^-N^*π*^). The notations *π* and *τ* are applied to differentiate the nitrogen atoms in the imidazole (McNaught and Wilkinson 1997)

Amongst various methods applied to study conformational properties of peptides and proteins, theoretical calculations based on short peptide models attract attention as native conformation depends considerably on local steric constraints and stabilising attractions (Culka et al. 2019; Culka and Rulíšek 2019, 2020; Řezáč et al. 2018; Abbenante et al. 1996; Brandt et al. 2011; Wahyudi and McAlpine 2015). In this study, using DFT methods, we examined the conformational properties of the simple molecular models, containing the imidazole-amino acid residue, where the imidazole ring is in place of the C-terminal amide group (Fig. 1). In consequence, the definition of the torsion angle *ψ* should be discussed. In a peptide chain, which consists only of standard amino acid residues, this angle is measured from the nitrogen atom at the N-terminus to the nitrogen atom at the C-terminus. If the imidazole ring is at the place of the C-terminal amide group, both imidazole nitrogen atoms can be included in the torsion angle *ψ*. In the studied molecules the methyl substituents at both termini imitate the peptide main chain; so that the symmetry of the imidazole ring is broken. According to histidine nomenclature, the nitrogen closer to the methyl group is denoted by *pros* ( ‘near’, abbreviated *π*) and another by *tele* (‘far’, abbreviated *τ*) (McNaught and Wilkinson 1997). In this way, the nitrogen *π* is part of the torsion angle *ψ*. In addition, the tautomers (*π* and *τ*) can be properly named and differentiated.

**Fig. 2:**
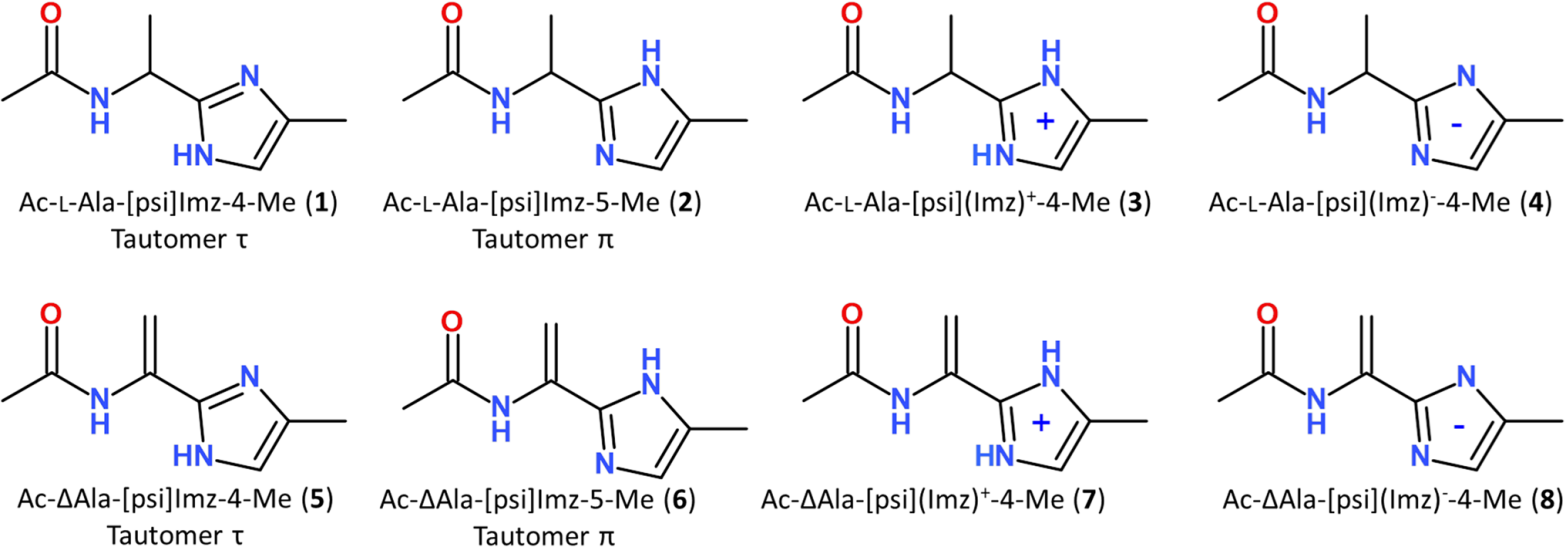
Schematic formula of the studied molecular models

The imidazole-alanine with the simplest methyl side chain was chosen for the studies. Additionally, it has been shown in our previous reports that the *α*,*β*-double bond in the side chain has the main influence on the conformation of the azole-containing residues (Siodłak et al. 2014b; Staś et al. 2016a, 2021). Hence, the imidazole-dehydroalanine residue, with the methylidene side chain, was also investigated. Because the imidazole structure is pH-dependent, not only two neutral forms (the tautomers *π* and *τ*) but also two other forms, with a positive or negative charge of the imidazole ring, were taken into account (Fig. 2).

## Computational details

The conformational properties of the following molecules were studied (Fig. **2**): *N*-[1-(4-methyl-1*H*-imidazol-2-yl)ethyl]acetamide (**1**), *N*-[1-(5-methyl-1*H*-imidazol-2-yl)ethyl]acetamide (**2**), *N*-[1-(4-methyl-1*H*-imidazol-3-ium-2-yl)ethyl]acetamide (**3**), *N*-[1-(4-methyl-1*H*-imidazol-3-id-2-yl)ethyl]acetamide (**4**), *N*-[1-(4-methyl-1*H*-imidazol-2-yl)ethenyl]acetamide, (**5**), *N*-[1-(5-methyl-1*H*-imidazol-2-yl)ethenyl]acetamide (**6**), *N*-[1-(4-methyl-1*H*-imidazol-3-ium-2-yl)ethenyl]acetamide (**7**), and *N*-[1-(4-methyl-1*H*-imidazol-3-id-2-yl)ethenyl]acetamide (**8**). According to the nomenclature rules, the NH takes precedence over the nitrogen N= in numbering atoms in the heteroring, in a consequence, in compound (**1**) the methyl substituent is at position 4 and in compound (**2**) at position 5. Conformational maps for all compounds were calculated in the gas phase, using partial optimization with the constrained torsion angles *φ* and *ψ*, changed with the step of 30 degrees, and in chloroform and water (single-point calculations) at M06-2X/6–31+G(d,p) level of theory (Zhao and Truhlar 2008). The method and basis set were chosen based on our previous studies (Siodłak et al. 2014b). To estimate the solvation effects on the conformations, calculations were also conducted using a self-consistent reaction field (SCRF) with the SMD method (Kang et al. 2011; Kang and Park 2014). The dehydroamino acids are achiral and each conformation (*φ*, *ψ*) has a related counterpart with the same energy but with opposite sign of torsion angles (− *φ*, − *ψ*) so that only half of the maps were calculated. For convenience, only the conformations located on the left side of the maps were analysed. The calculations were performed for the molecules with the *trans-*amide bond. Full optimization was performed for all found local minima on the maps using a bigger basis set, 6–311++ G(d,p). Frequency analyses were carried out to verify the nature of the minimum state of all stationary points and to calculate the zero-point vibrational energies (ZPVEs). The Gaussian 16 package was used (Frisch et al. 2016).

The expected population (*p*) of the conformations at temperature 300 K (where *RT* = 0.595 kcal/mol) were calculated (Hudáky and Perczel 2008; Hruby et al. 1997). The nomenclature of conformations is based on the Scarsdale nomenclature (Scarsdale et al. 1983; Hudáky et al. 2004), however, notations *π* and *τ* were added to differentiate the conformation for both tautomers (McNaught and Wilkinson 1997). Imidazole has various abbreviations such as *Imi, Imd, Imz* (All Acronyms. Imidazole 2022), we decided to apply *Imz*. The parameters created within the studied residues intramolecular hydrogen bonds and dipole–dipole interactions are included in Tables S1–S4 in the Supplementary Information. The NBO analysis (Weinhold and Landis 2001) was performed using the same method and basis set as mentioned before (Table S5).

## Results

### Imidazole-alanine (1 and 2)

**Fig. 3:**
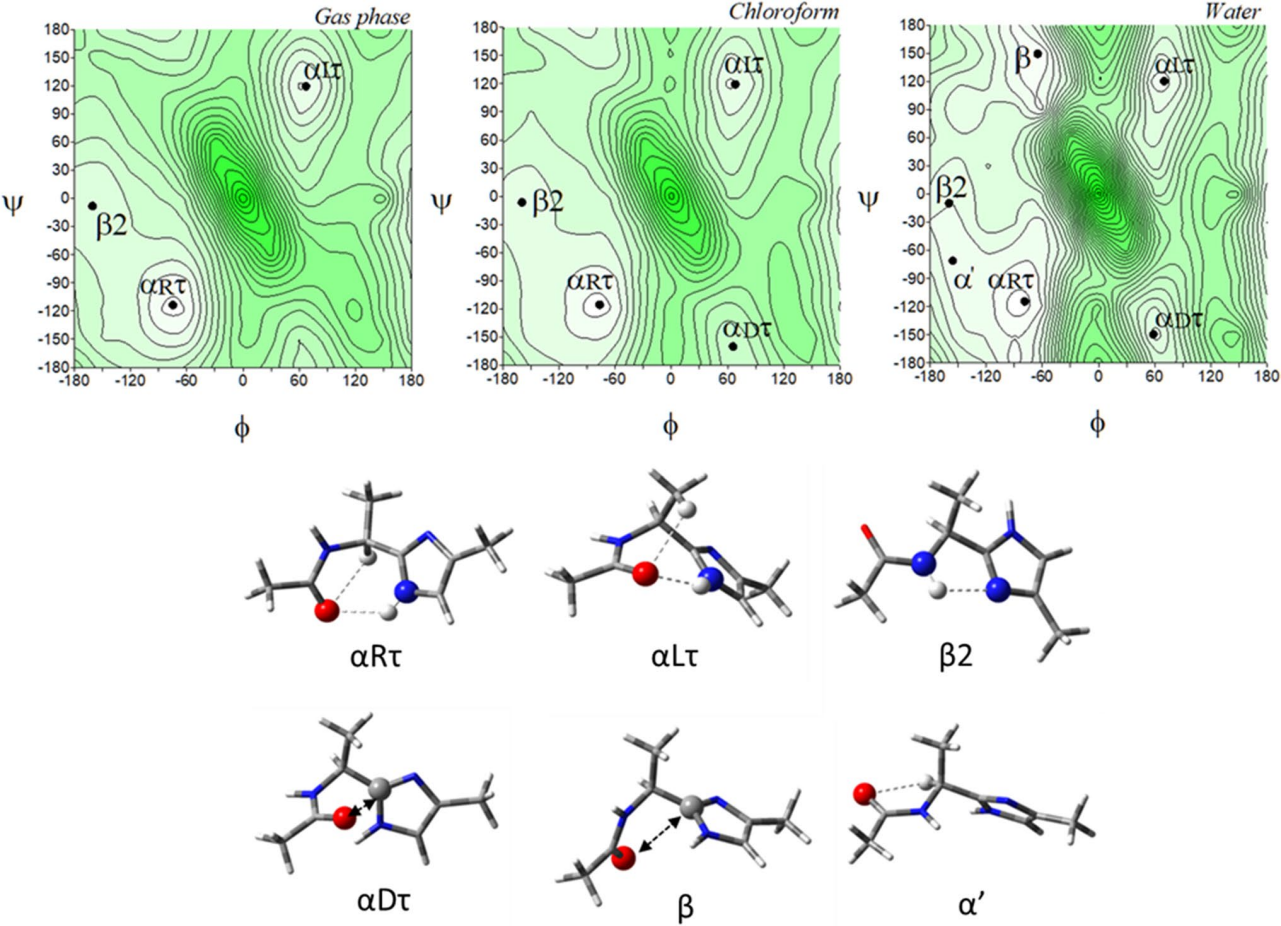
The potential energy surfaces *E* = *f*(*φ*,*ψ*) of Ac-l-Ala-[psi]Imz-4-Me (**1**) calculated at M06-2X/6–31+G(d,p) method in the gas phase, chloroform, and water environments. Energy contours are plotted every 1 kcal/mol. The darker colour indicates the high in energy regions and the lighter—low in energy regions. Below maps are the conformations of Ac-l-Ala-[psi]Imz-4-Me (**1**) optimised in water with the most important electrostatic interactions (◄⋯►) and hydrogen bonds (⋯) created within the residue

The potential energy surfaces of the imidazole-alanine tautomer *τ*, Ac-l-Ala-[psi](Imz)-4-Me (**1**), in three environments of different polarity: gas phase, chloroform, and water are shown in (Fig. 3). For the isolated molecule, mimicked by the gas phase, the map presents three local minima, which correspond to the conformations: *αRτ*, *αLτ*, and *β*2. The global minimum is occupied by the right-handed helical conformation *α*R*τ* (*φ*, *ψ* = − 74.8°, − 113.7°) (Table 1). This conformation is stabilized by two intramolecular hydrogen bonds created within the studied residue: N^*τ*^-H···O=C and C^*α*^-H···O=C (Fig. 3). In both cases, the acceptor of the hydrogen bond is the oxygen atom of the N-terminal amide group. For the N^*τ*^-H···O=C hydrogen bond the donor is the imidazole nitrogen atom *τ*. The second in energy order, the conformation *αLτ* (*φ*, *ψ* = 67.1°, 119.6°) has the values of the torsion angles *φ* and *ψ* almost opposite to the conformation *αRτ*, so that it is stabilized by similar hydrogen bonds. However, due to the configuration of the C^*α*^ (L-chirality), instead of the C^*α*^-H···O = C hydrogen bond, a weaker C^*β*^-H···O=C interaction is formed. The highest in energy is the conformation *β*2 (*φ*, *ψ* = − 160.9°, − 8.0°). It is stabilized by the intramolecular hydrogen bond N–H···N^*π*^ formed between the hydrogen atom of the N-terminal amide group and the nitrogen atom *π* of the imidazole. The relative differences in energy, expressed also by the expected population (*p*) of the conformers indicate that almost all molecules adopt the right-handed helical conformation *αRτ*. The predicted population of the two others is very small (Table 1). Nevertheless, as can be seen, all possible conformations are stabilised by the hydrogen bond created within the studied residue in which, the nitrogen atoms of the imidazole ring are involved.

**Table 1:** Selected torsion angles (°) of local minima for Ac-l-Ala-[psi]Imz-4-Me (**1**) and Ac-l-Ala-[psi]Imz-5-Me (**2**) optimized at M06-2X/6–311++G(d,p) method, their relative energies (ΔE) and the relative energy difference between tautomers (ΔE*τ*/*π*) in kcal/mol, and population (*p*)

Ac-l-Ala-[psi]Imz-4-Me (1)							Ac-l-Ala-[psi]Imz-5-Me (2)						
Conformer	*φ*	*ψ*	*E* [a.u.]	Δ*E*	Δ*Eτ/π*	*p* [%]	Conformer	*φ*	*ψ*	*E* [a.u.]	Δ*E*	Δ*Eτ/π*	*p* [%]
*Gas Phase*							*Gas Phase*						
*α*R*τ*	− 74.8	− 113.7	− 346,591.1629	0.00	0.00	53.45	C7eq	− 74.8	66.3	− 346,590.9812	0.00	0.18	39.39
*α*L*τ*	67.1	119.6	− 346,589.5201	1.64	1.64	3.38	C7ax	67.1	− 64.2	− 346,589.5096	1.47	1.65	3.33
*β*2	− 160.9	− 8.0	− 346,587.9694	3.19	3.19	0.25	C5	161.0	174.8	− 346,587.8418	3.14	3.32	0.20
					**Sum *p***	**57.09**						**Sum *p***	**42.91**
*Chloroform*							*Chloroform*						
*α*R*τ*	− 76.7	− 115.6	− 346,603.6196	0.00	0.22	36.80	C7eq	− 77.0	64.4	− 346,603.8358	0.00	0.00	52.92
*α*L*τ*	68.3	119.4	− 346,602.1772	1.44	1.66	3.26	C7ax	68.3	− 64.2	− 346,602.4303	1.41	1.41	4.99
*β*2	− 159.7	− 6.5	− 346,601.4955	2.12	2.34	1.04	C5	− 159.4	172.5	− 346,601.4301	2.41	2.41	0.93
*α*D*τ*	65.9	− 160.2	− 346,599.2365	4.38	4.60	0.02	*α*L*π*	65.0	24.4	− 346,599.2519	4.58	4.58	0.02
					**Sum *p***	**41.13**						**Sum *p***	**58.87**
*Water*							*Water*						
*α*R*τ*	− 79.0	− 114.3	− 346,603.6149	0.00	0.45	14.11	C7eq	− 79.1	65.0	− 346,604.0618	0.00	0.00	29.88
*β*	− 65.1	149.6	− 346,603.4702	0.14	0.59	11.06	*α*R*π*	− 63.9	− 34.5	− 346,603.8546	0.21	0.21	21.10
*β*2	− 159.9	− 9.6	− 346,602.7493	0.87	1.31	3.30	C7ax	70.0	− 63.5	− 346,603.0224	1.04	1.04	5.21
*α*L*τ*	70.1	120.5	− 346,602.6386	0.98	1.42	2.74	C5	− 160.0	173.4	− 346,602.9620	1.10	1.10	4.71
*α*'	− 156.0	− 70.9	− 346,602.3645	1.25	1.70	1.73	*β*2*π*	− 154.9	94.0	− 346,602.7599	1.30	1.30	3.36
*α*D*τ*	58.7	− 149.3	− 346,601.9275	1.69	2.13	0.83	*α*L*π*	57.8	37.4	− 346,602.4450	1.62	1.62	1.98
					**Sum *p***	**33.76**						**Sum *p***	**66.24**

For the environment of low polarity, mimicked by chloroform solvent, the conformations are maintained in the same order as for the isolated molecule (Table 1). Change of the environment from the gas phase to chloroform caused less than 2° adjustment of the torsion angles. Additionally, the conformation *αDτ* (*φ*, *ψ* = 65.9°, − 160.2°) appears, stabilized by the dipole–dipole interaction created by the carbonyl group of the amide bond (Allen et al. 1998). Its relative energy is high, and thus estimated population is around 0.02%.

The biggest conformational changes occur when the polar water environment is mimicked. In this solvent, the imidazole-alanine (**1**) adopts six conformations: *αRτ*, *β*, *β*2, *αLτ*, *α*’, and *αDτ* (Fig. 3). Two new conformations, *β* (*φ*, *ψ* = − 65.1°, − 149.6°) and *α*’ (*φ*, *ψ* = − 156.0°, − 70.9°) appear. The conformations *β* is stabilized by dipole–dipole interaction between the amide bond and imidazole and becomes second in the energy order. In the conformation *α*’ the C^*α*^-H···O=C hydrogen bond is formed. The energy difference between the lowest and highest in energy conformations considerably diminishes to 2.13 kcal/mol. The left side of the map becomes flattered as compared to the maps for the isolated molecule and the weakly polar environments. This shows that in the polar environment the conformational freedom of the compound (**1**) should be wider. Interestingly, the environment does not influence much the geometry of the conformations *αRτ*, *αLτ*, and *β*2. Their torsion angles *φ* and *ψ* change merely by a few degrees. The most considerable changes around 10° are observed for the conformation *αDτ* stabilized by dipole interactions created by the amide oxygen atom and the imidazole C2 atom. This indicates considerable stability of the hydrogen bonds created by the nitrogen atoms of the imidazole ring. The lowest in energy is still the conformation *αRτ*, which means that the stabilizing forces within the residue are maintained even in the polar environment, where they compete with intermolecular interactions. The internal dipole–dipole interactions are responsible for the appearance of the conformations *αDτ*, *β*, and *α*’. To sum up, the most preferable conformation for tautomer *τ* is the right-handed helical conformation *αRτ*, regardless of the simulated environment.

**Fig. 4:**
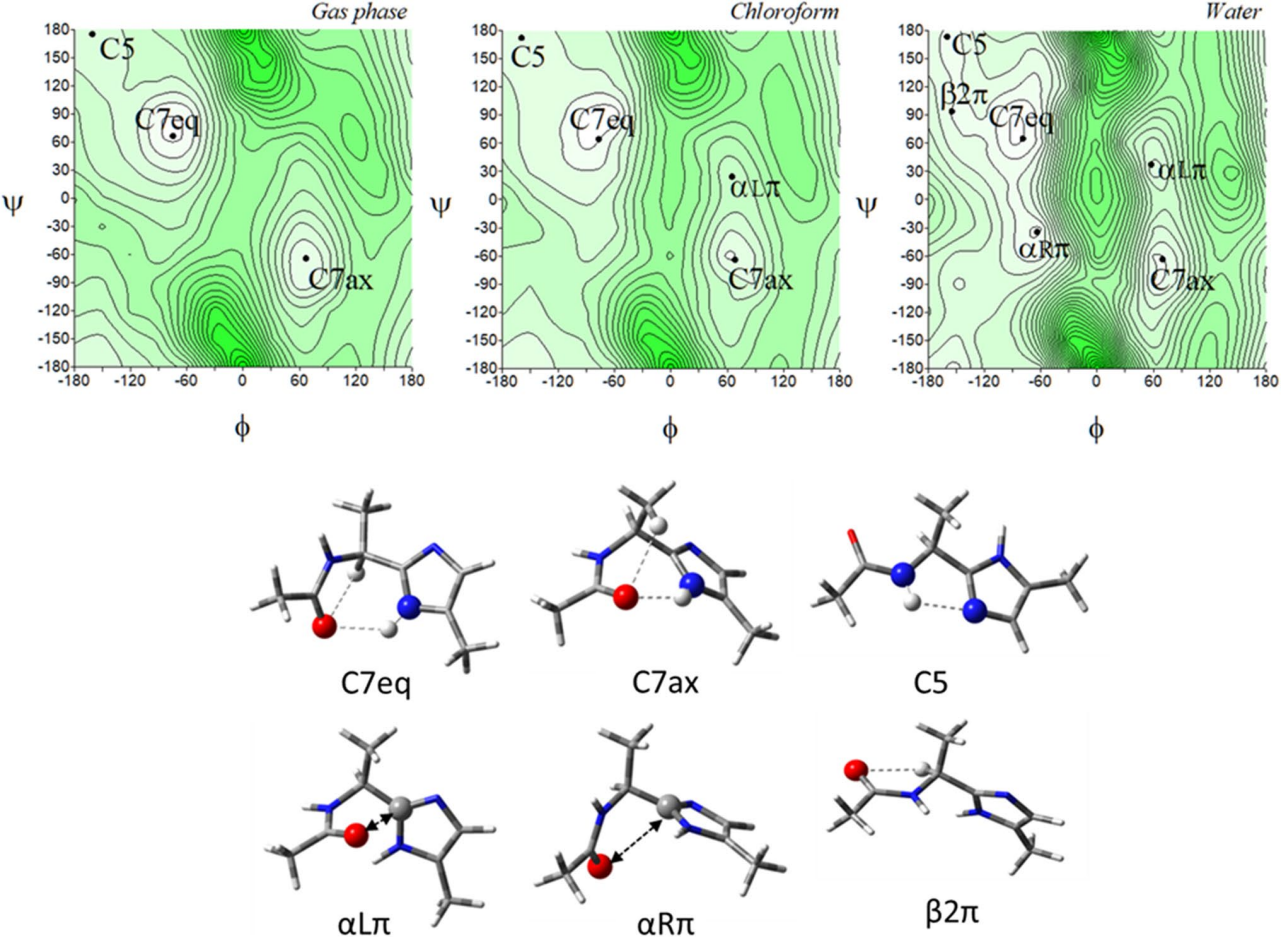
The potential energy surfaces *E* = *f*(*φ*,*ψ*) for Ac-l-Ala-[psi]Imz-5-Me (**2**) calculated at M06-2X/6–31+G(d,p) method in the gas phase, chloroform, and water. Energy contours are plotted every 1 kcal/mol. The darker colour indicates the high in energy regions and the lighter—low in energy regions. Below maps are the conformations optimised in water with the most important electrostatic interactions (◄⋯►) and hydrogen bonds (⋯) created within the residue

Figure 4 presents the potential energy surfaces of the imidazole-alanine tautomer *π*, Ac-l-Ala-[psi](Imz)-5-Me (**2**), in the three environments of different polarity together with their conformations. For the isolated molecule, the map presents three local minima, which correspond to the conformations: C7eq, C7ax, and C5. The global minimum is occupied by the conformation C7eq (*φ*, *ψ* = − 74.8°, 66.3°) (Table 1), stabilized by two intramolecular hydrogen bonds within the studied residue, N^*π*^-H···O=C and C^*α*^-H···O=C, created by the N-terminal amide group. For the N^*π*^-H···O=C hydrogen bond the donor is the imidazole nitrogen atom *π*. The second in energy order, the conformation C7ax (*φ*, *ψ* = 67.1°, − 64.2°) is stabilised also by the N^*π*^-H···O=C hydrogen bond as well as minor C^*β*^-H···O=C interaction. The third in energy, the conformation *β*2 (*φ*, *ψ* = 161.0°, 174.8°) is stabilized by the N–H···N^*τ*^ hydrogen bond where the acceptor is the imidazole nitrogen atom *τ*. The relative differences in energy indicate that the conformation C7eq will be mainly adopted. In the low polar chloroform environment, the conformation *αLπ* (*φ*, *ψ* = 65.0°, 24.4°) appears, stabilized by the dipole–dipole interaction created by the carbonyl group of the amide bond. More polar water environment results in the appearance of two new conformations, *αRπ* (*φ*, *ψ* = − 63.9°, − 34.5°) and *β2π* (*φ*, *ψ* = − 154.9°, 94.0°). The conformations are stabilised, respectively, by the dipole–dipole interaction between the amide bond and the imidazole as well as the C^*α*^-H···O=C hydrogen bond. As can be seen, the most preferable conformation for the tautomer *π* is the conformation C7eq, regardless of the simulated environment.

A comparison of conformational preferences of both tautomers, *τ* (**1**) and *π* (**2**) shows a specific symmetry (Fig. S2). Although the tautomer *π* (**2)** has the all-new suite of conformations, each conformation has the corresponding conformation of the tautomer *τ* in terms of relative energy: *α*R*τ* (**1**) vs C7eq (**2**), *β* (**1**) vs *α*R*π* (**2**), *β*2 (**1**) vs C5 (**2**), *α*L*τ* (**1**) vs C7ax (**2**), *α*’ (**1**) vs *β*2 (**2**), and *α*D*τ* (**1**) vs *α*L*π* (**2**) (compare Figs. 3 and 4). The analysis of their geometry, by comparison of the values of torsion angles *φ* and *ψ*, shows that the torsion angle *φ* is almost the same for these pairs of conformations (Table 1). For example, *φ* = − 74.8° in *α*R*τ* (1) and C7eq (2), while the value of the torsion angles *ψ* differs roughly by ± 180°. It is the result of the imidazole ring rotation caused by the migration of the hydrogen atom, between the nitrogen atoms, from *τ* to *π*. The change of tautomer causes that each pair of conformations is stabilised by the same internal forces. The most stable conformations, *α*R*τ* (**1**) and C7eq (**2**) are maintained by the N^Imz^-H···O=C hydrogen bond. Despite the similarity between the tautomers, the methyl substituent at the imidazole ring has a different position in space. It represents the continuation of the main chain in the peptide so that the choice of tautomer will have a tremendous impact on the conformation adopted by the peptide. The stability of the lowest in energy conformations, *α*R*τ* (**1**) and C7eq (**2**), regardless of the environment simulated and the same stabilising intramolecular forces created within the analysed structural motif enables to predict of a tendency towards a given tautomer depending on the polarity of environment. Both conformations have similar energy (Δ*Eτ*/*π*), which does not exceed 0.45 kcal/mol (Table 1), so they should occur in equilibrium. For a non-polar environment, more stable is the conformation *α*R (**1**), thus the population of the tautomer *τ* is higher than that of the tautomer *π* (53% vs 39%). As the polarity of the environment increases, the preference is shifted towards the tautomer *π*.

There is no information so far about the crystal structure of Ala-[psi]Imz residue. However, there are data about macrocycle peptide synthesised by Haberhauer and co-workers (Haberhauer et al. 2005) where Val-[psi]Imz residue is part of the macrocyclic chain as the analogue of the tautomers *τ* (**1**) with the torsion angles *φ*, *ψ* = − 129.4°, 142.6° and *φ*, *ψ* = − 129.4°, − 39.2°. Such angles correspond to the conformations C5 and *β*2, which are predicted to be higher in energy. It should be stressed, however, that the macrocycle consisting of four amino acid residues imposes some steric constraints.

A comparison of the studied imidazole-alanine to the oxazole-alanine (Siodłak et al. 2014a) and the thiazole-alanine (Staś et al. 2021) shows that the set of conformations is similar, which results from structural similarity. However, the energy order, and thus, conformational preferences are different. For the thiazole analogue, Ala-[psi](Tzl), in the gas phase the lowest in energy is the conformation *β*2 (*φ*, *ψ* ~ − 160°, − 6°). As the polarity of the environment increases, in chloroform and water, a tendency towards the conformation *β* (*φ*, *ψ* ~ − 75°, 160°) can be seen. For the oxazole analogue, Ala-[psi](Ozl), the preferences towards the conformation *β*2 (*φ*, *ψ* ~ − 155°, − 10°) is observed not only in the gas phase but also in weakly polar chloroform. The conformation *β* (*φ*, *ψ* ~ − 60°, 145°) prevails in the water environment. These conformations are stabilised by the N–H···N hydrogen bond created within the residue when the nitrogen atom of the heteroaromatic ring is an acceptor of dipole interactions. The conformation *α*R is not seen for the Ala-[psi](Ozl) residue or it is of much higher energy for Ala-[psi](Tzl), which results from much weaker dipole stabilisation. The shapes of the conformational maps indicate that Ala-[psi](Imz) is the least flexible and has the best-defined minima and is least dependent on the simulated environment.

## Imidazolium-alanine (3)

**Fig. 5:**
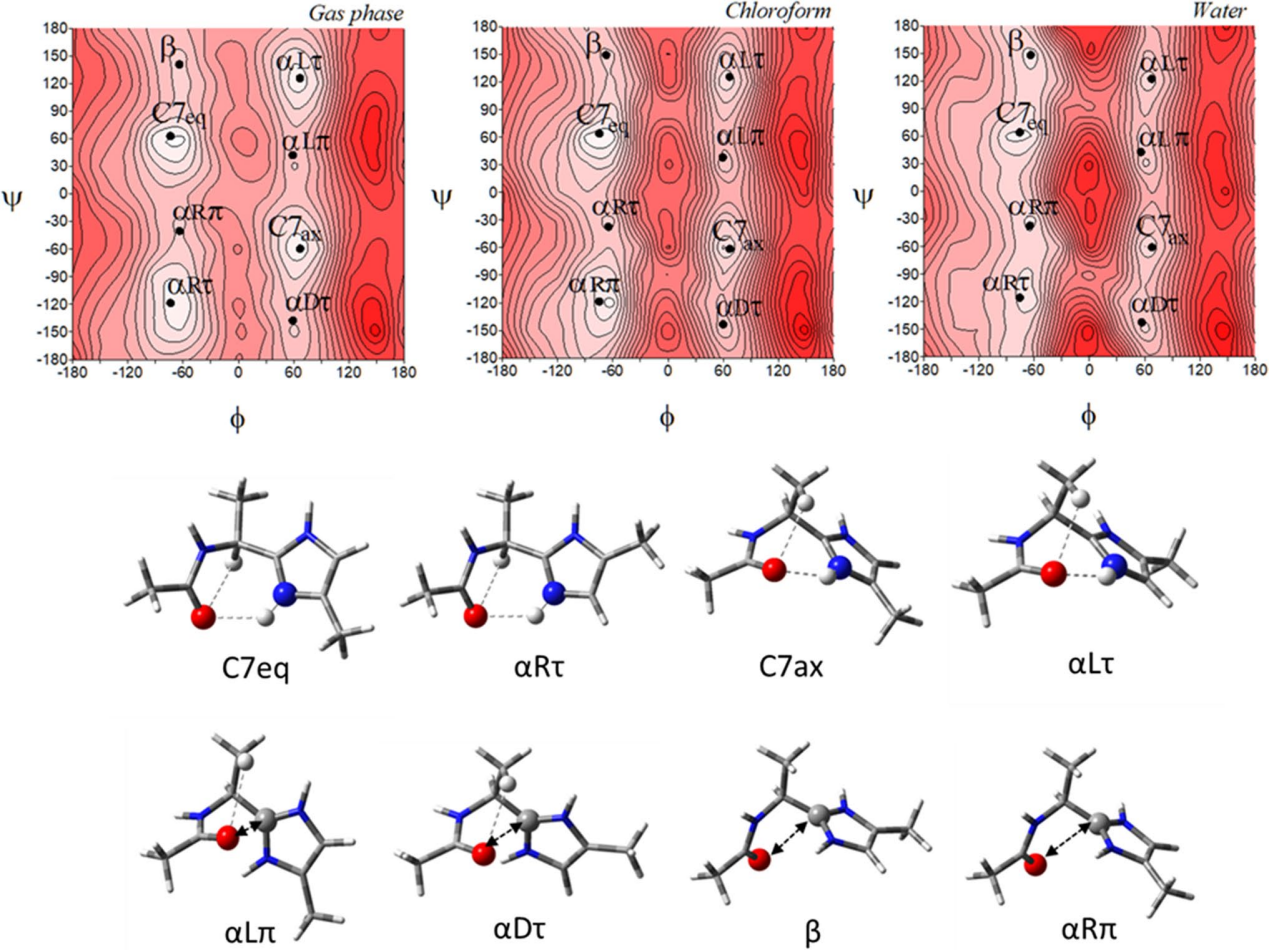
The potential energy surfaces *E* = *f*(*φ*,*ψ*) for Ac-l-Ala-[psi](Imz)^+^-4-Me (**3**) calculated at M06-2X/6–31+G(d,p) method in the gas phase, chloroform, and water. Energy contours are plotted every 1 kcal/mol. The darker colour indicates the high in energy regions and the lighter—low in energy. Below maps are the conformations optimised in water with the most important electrostatic interactions (◄⋯►) and hydrogen bonds (⋯) created within the residue

Imidazole ring has the properties to accept a proton and, as a consequence, to gain a positive charge. Therefore, the residue with the positive charge was also studied. The potential energy surfaces for the protonated imidazolium-alanine, Ac-l-Ala-[psi](Imz)^+^-4-Me (**3**), in the gas phase, chloroform and water show eight minima, regardless of the polarity of the environment (Fig. 5). The conformations correspond to those found for the neutral tautomers *τ* (1) and *π* (2). The conformations *α*R*τ* (*φ*, *ψ* = − 73.8°, − 118.6°), *α*L*τ* (*φ*, *ψ* = 66.9°, 126.0°), *α*D*τ* (*φ*, *ψ* = 59.1°, − 137.6°), and *β* (*φ*, *ψ* = − 64.2°, 140.9°) are similar to those of the tautomer *τ*. The other four conformations: C7eq (*φ*, *ψ* = − 73.9°, 63.0°), C7ax (*φ*, *ψ* = 67.0°, − 59.5°), *α*L*π* (*φ*, *ψ* = 59.2°, 42.3°), and *α*R*π* (*φ*, *ψ* = − 64.1°, − 40.2°) are similar to those of the tautomer *π*. The conformations are stabilised by the same intramolecular interactions as in the case of the tautomers *τ* (**1**) and *π* (**2**). As could be expected, the lowest energy and the highest populations reveal the conformations *α*R*τ* and C7eq (Table 2). Both these conformations have the same value of the torsion angle *φ* and the torsion angle *ψ* differs by 180°. The imidazole ring becomes symmetrical after protonation, both nitrogen atoms become the donor of the hydrogen bond and the methyl substituent does not interfere with the formed internal interactions. The conformations are stabilised by the N^*π*^-H···O and C^*α*^-H···O hydrogen bonds created within the residue, and therefore, the energy difference between them is very small (ΔE ~ 0.2 kcal/mol). The presented study predicts a conformational equilibrium and the choice of conformation will depend on distal interactions, but not the interactions created within the studied imidazolium-alanine residue (**3**). The next in energy order, for the isolated molecule and in a low polar environment, are the pair of conformations, C7ax and *α*L*τ*. They are also stabilised by the N^*π*^-H···O hydrogen bonds, but the chirality of the carbon *α* results in the lack of the C^*α*^-H···O hydrogen bond so their energy is a little higher (Δ*E* ~ 1.0–1.5 kcal/mol). The increase of the polarity of the environment results in a further decrease in energy difference between the conformations, especially for the conformations *β*, *α*L*τ*, *α*D*τ*, and *α*L*π*, which are stabilised by dipole interactions. The presented results indicate that the environment does not influence the geometry of conformations of the imidazolium-alanine, however, it affects their relative energy.

**Table 2:** Selected torsion angles (°) of local minima for Ac-l-Ala-[psi](Imz)^+^-4-Me (**3**) optimized at M06-2X/6–311++G(d,p) method, their relative energies (ΔE) and the relative energy difference between tautomers (Δ*Eτ*/*π*) in kcal/mol, and population (*p*)

Ac-l-Ala-[psi](Imz)^+^-4-Me (3)					
Conformer	*φ*	*ψ*	*E* [a.u.]	Δ*E*	*p* [%]
*Gas Phase*					
C7eq	− 73.9	63.0	− 552.5016	0.00	49.71
*α*R*τ*	− 73.8	− 118.6	− 552.5012	0.19	35.84
C7ax	67.0	− 59.5	− 552.4998	1.09	8.00
*α*L*τ*	66.9	126.0	− 552.4995	1.23	6.27
*α*L*π*	59.2	42.3	− 552.4950	4.08	0.05
*α*D*τ*	59.1	− 137.6	− 552.4950	4.13	0.05
*β*	− 64.2	140.9	− 552.4951	4.18	0.04
*α*R*π*	− 64.1	− 40.2	− 552.4950	4.24	0.04
*Chloroform*					
*α*R*τ*	− 74.7	− 118.6	− 552.5808	0.00	51.62
C7eq	− 74.8	64.2	− 552.5809	0.15	40.26
C7ax	67.1	− 61.4	− 552.5786	1.63	3.34
*α*L*τ*	66.9	125.8	− 552.5784	1.71	2.93
*α*L*π*	59.5	38.2	− 552.5767	2.64	0.61
*α*R*π*	− 64.9	− 37.2	− 552.5772	2.68	0.57
*β*	− 67.4	149.5	− 552.5771	2.83	0.45
*α*D*τ*	59.9	− 143.5	− 552.5765	3.25	0.22
*Water*					
*α*R*τ*	− 75.6	− 115.5	− 552.5923	0.00	33.19
C7eq	− 75.9	64.2	− 552.5924	0.07	29.59
*α*R*π*	− 62.4	− 38.4	− 552.5919	0.57	12.69
*β*	− 63.9	148.2	− 552.5918	0.64	11.33
*α*L*π*	55.9	42.8	− 552.5906	1.09	5.28
C7ax	67.9	− 60.6	− 552.5902	1.42	3.07
*α*D*τ*	56.9	− 142.2	− 552.5904	1.61	2.23
*α*L*τ*	67.6	122.4	− 552.5901	1.51	2.63

## Imidazolide-alanine (4)

**Fig. 6:**
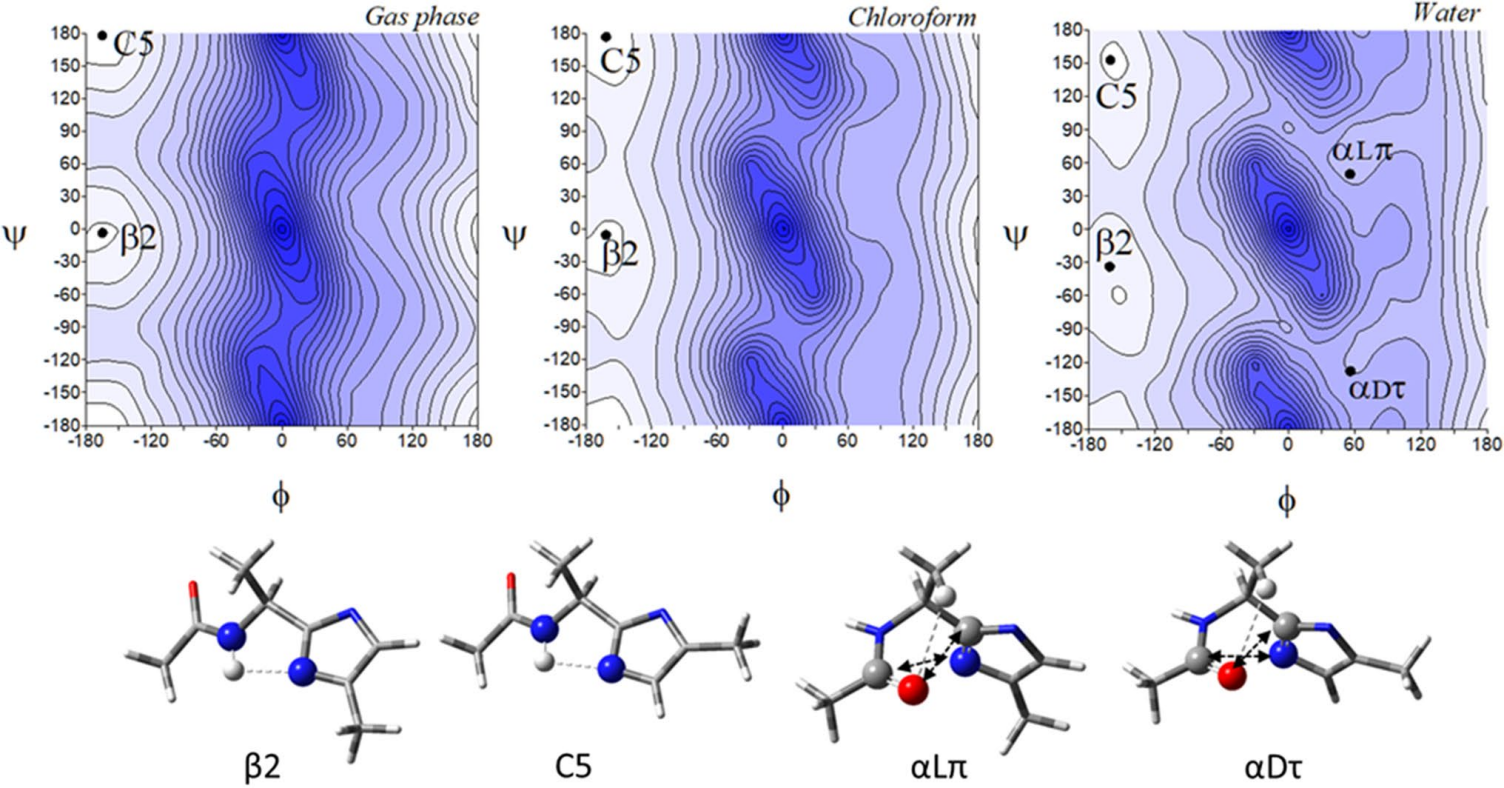
The potential energy surfaces *E* = f(*φ*,*ψ*) for Ac-l-Ala-[psi](Imz)^−^-4-Me (**4**) calculated at M06-2X/6–31+G(d,p) method in the gas phase, chloroform, and water. Energy contours are plotted every 1 kcal/mol. The darker colour indicates the high in energy regions and the lighter—low in energy. Below maps are conformations optimised in water with the most important electrostatic interactions (◄⋯►) and hydrogen bonds (⋯) created within the residue

The ability of deprotonation of the imidazole ring enables the molecule to be negatively charged. Thus, we also decided to check how the deprotonation influences the imidazole-alanine conformation. The conformational maps of Ac-l-Ala-[psi](Imz)^−^-4-Me (**4**) (Fig. 6) present in the gas phase only two minima, which correspond to the semi-extended conformation *β*2 (*φ*, *ψ* = − 165.2°, − 3.4°) and the extended conformation C5 (*φ*, *ψ* = − 165.3°, 178.1°). Both conformations are stabilized by the N–H···N^Imz^ hydrogen bond formed between the hydrogen atom of the amide group and one of the nitrogen atoms of imidazole acting as the hydrogen bond acceptor. The energy difference between these two conformations for the isolated molecule is small, does not exceed 0.21 kcal/mol, and the population ratio is roughly around 50:50% (Table 3). The conformations differ by the rotation of the torsion angle *ψ* by half a turn, resulting in the different position of ring substituent—the methyl group at position 4. The substituent, which represents the potential peptide main chain, does not cause any critical barrier, so it will not constraint the conformational freedom. Also, it does not create any internal stabilizing interaction within the studied imidazolide-alanine residue (**4**). The energy profit gained from the creation of the N–H···N hydrogen bond by the nitrogen atoms of the imidazole is the same. A good example of that is the energy difference between the conformations C5 and *β*2 in the water polar environment (0.06 kcal/mol) as well as those for the conformations *β* vs *α*L (0.11 kcal/mol). The parameters for the hydrogen bond are almost the same (Table S1). An increase of environment polarity does not result in considerable conformational changes. In water, the tendency to adopt the conformations C5 and *β*2 is still very strong, however, a very small percentage of another two conformations occur, *α*L*π* (*φ*, *ψ* = 56.0°, 50.1°) and *α*D*τ* (*φ*, *ψ* = 56.3°, − 128.1°).

**Table 3:** Selected torsion angles (°) of local minima for Ac-l-Ala-[psi](Imz)^−^-4-Me (**4**) optimized at M06-2X/6–311++G(d,p) method, their relative energies (Δ*E*) and the relative energy difference between tautomers (Δ*Eτ/π*) in kcal/mol, and population (*p*)

Ac-l-Ala-[psi](Imz)^−^-4-Me (4)					
Conformer	*φ*	*ψ*	*E* [a.u.]	Δ*E*	*p* [%]
*Gas Phase*					
C5	− 165.3	178.1	− 551.5603	0.00	58.67
*β*2	− 165.2	− 3.4	− 551.5600	0.21	41.33
*Chloroform*					
*β*2	− 162.1	− 5.4	− 551.6351	0.00	50.46
C5	− 162.1	176.8	− 551.6353	0.01	49.54
*Water*					
C5	− 160.7	153.1	− 551.6555	0.00	51.87
*β*2	− 161.3	− 33.7	− 551.6555	0.06	46.86
*α*L*π*	56.0	50.1	− 551.6512	2.57	0.69
*α*D*τ*	56.3	− 128.1	− 551.6510	2.68	0.58

The lack of the imidazole hydrogen atom changes the electron distribution in the ring (Fig. S3, Table S5) and the intermolecular interaction pattern, which influences the conformational preferences, as compared to the neutral forms (**1**) and (**2**) as well as positively charged form (**3**). The imidazole nitrogen atoms for (**4**) are of the same type, both can create interaction with the same energy. As a consequence, the conformations correspond to those found in both neutral tautomers *τ* (**1**) and *π* (**2**). The conformations *β*2 and *α*D*τ* are similar to those of the tautomer *τ*. The other two conformations, C5 and *α*L*π* are similar to those of the tautomer *π*. Also, the conformations are stabilised by the same intramolecular interactions as in the case of the tautomers. Nevertheless, as the main stabilizing force is the internal N–H···N^Imz^ hydrogen bond, the conformations C5 and *β*2 with the torsion angle *φ* of about -162° are stable regardless of the environment, which determines the unique conformational properties of the studied imidazolide-alanine residue (**4**).

## Imidazole-dehydroalanine (5) and (6)

In the world of natural compounds, the residues with the double bond between the carbon atoms *α* and *β* in the side chain can be also found (Siodłak 2015). The carbon *α* does not have chirality, which influences the conformational profile of the dehydroamino acid residue. The double bond C*α* = C*β* can potentially conjugate with the imidazole ring, thus, the conformational properties of this artificial residue were also studied.

**Fig. 7:**
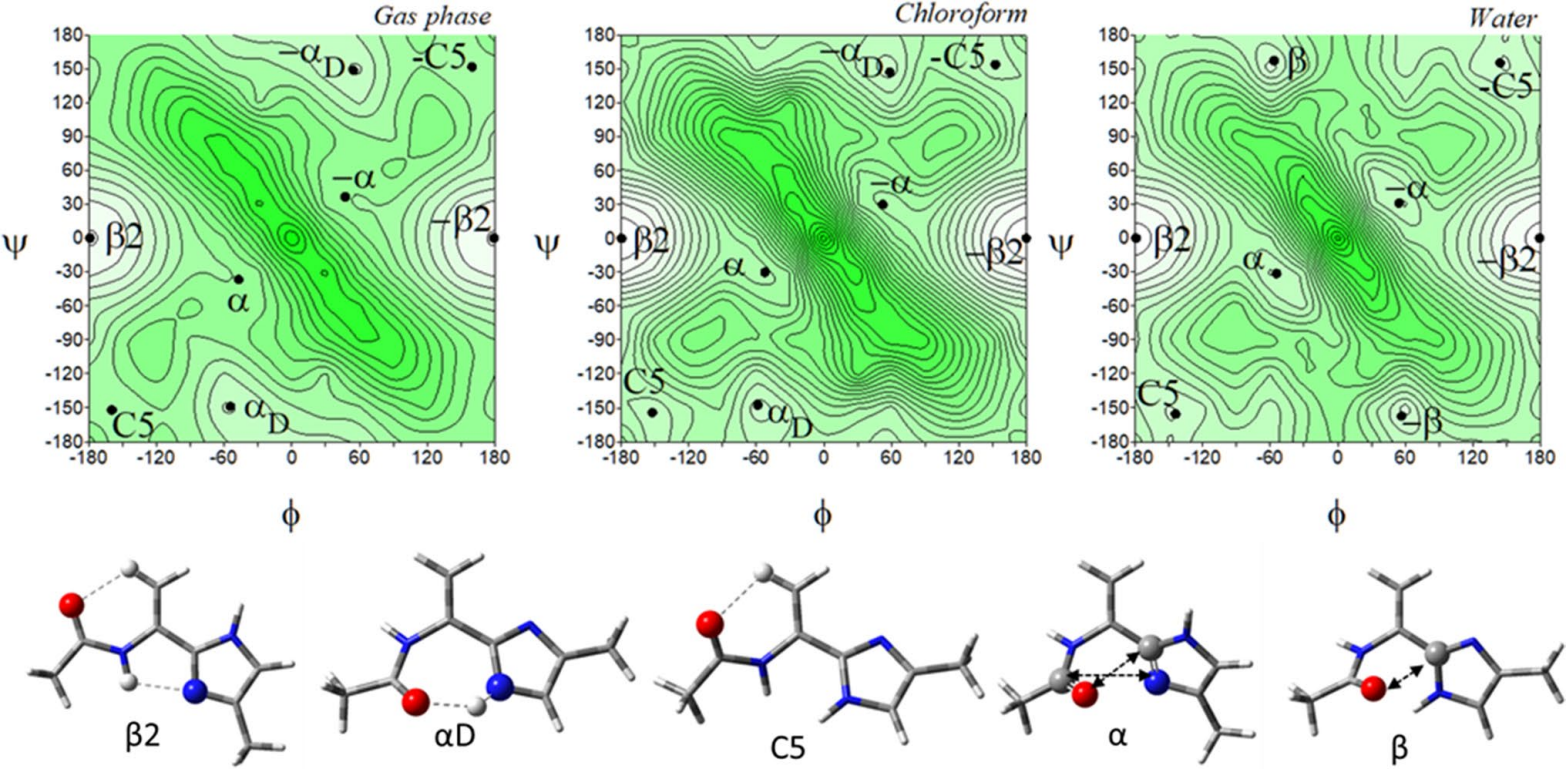
The potential energy surfaces *E* = *f*(*φ*,*ψ*) for Ac-ΔAla-[psi]Imz-4-Me (**5**) calculated at M06-2X/6–31+G(d,p) method in the gas phase, chloroform, and water. Energy contours are plotted every 1 kcal/mol. The darker colour indicates the high in energy regions and the lighter—low in energy. Below maps are conformations optimised in water with the most important electrostatic interactions (◄⋯►) and hydrogen bonds (⋯) created within the residue

The conformational maps of the tautomer *τ* of the imidazole-dehydroalanine, Ac-ΔAla-[psi]Imz-4-Me (**5**) show four pairs of energy minima corresponding to the conformations: *β*2, *α*D, C5, and *α* (Fig. 7). Their mirror conformations in the maps, which means the conformations with the same energy value but the opposite sign of torsion angles, are marked as -*β*2, -*α*D, -C5, and -*α*. As can be seen, the energy potential surfaces are symmetrical, due to the lack of the asymmetric *α*-carbon atom in the molecule. Hence, for clarity, the left side of the maps was chosen for the analysis, which also reduced the number of symbols of the conformations, excluding the mirror counterparts.

The global potential energy minimum is occupied by the conformation *β*2 (*φ*, *ψ* = − 179.3° ± 0.6°, 0.2° ± 0.2°), regardless of the mimicked environment (Table 4). As for the imidazole-alanine residue (**1**), the conformation *β*2 is stabilized by the N–H···N^*π*^ hydrogen bond. Nevertheless, due to the hybridisation sp^2^ of the carbon *α*, the bonds are shorter and the parameters of this hydrogen bond are different (better) (Table S1 and **S2**). Also, the hydrogen bond C^*β*^-H···O is formed. Moreover, due to the value torsion angles ensuring the alignment of the carbon skeleton in the plane, the additional stabilization force occurs, the *π*-electron cross-conjugation between the amide group, double bond, and imidazole ring. As a result, the conformation *β*2 is very stable and has a very high population. Other conformations, *α*D (*φ*, *ψ* ~ − 54.4°, − 148.9°), C5 (*φ*, *ψ* ~ − 159.8°, − 151.9°), and *α* (*φ*, *ψ* ~ − 47.3°, − 36.6°) are stabilised by single hydrogen bond or dipole interaction, however, they have marginal population; in the gas phase and chloroform it is less than 0.2% (combined) and in water is a little bit higher around 1–2% each. This indicates that concomitant interactions, the *π*-electron conjugation and the intramolecular hydrogen bonds, N–H···N^*π*^ and C^*β*^-H···O, considerably lower the energy of the molecule. In polar solvent, the conformation *α*D vanishes and the conformation *β* (*φ*, *ψ* = − 56.7°, 157.4°) appears in its place. A comparison of these conformations shows that the value of the torsion angle *φ* remains almost the same, only the value of the torsion angle *ψ* changes. The rotation around the torsion angle *ψ* about 60° results in the change of stabilised force within the residue, from characteristic for peptide N–H···O hydrogen bond, which involves seven atoms (the conformation *α*D) to dipole–dipole interactions (the conformation *β*). This change makes the residue more open for intermolecular hydrogen bonds, in which the imidazole N–H group can participate. Typically, the energy difference between the conformations decreases with the increasing polarity of the environment. Minima are well defined on the maps. Interestingly, the geometry of the conformations changes only about 2° and in the most extreme case 7°, so the conformational freedom seems to be considerably limited.

**Table 4:** Selected torsion angles (°) of local minima for Ac-ΔAla-[psi]Imz-4-Me (**5**) and Ac-ΔAla-[psi]Imz-5-Me (**6**), optimized at M06-2X/6–311++G(d,p) method, their relative energies (ΔE) and the relative energy difference between tautomers (Δ*Eτ/π*) in kcal/mol, and population (*p*)

Ac-ΔAla-[psi]Imz-4-Me (5)							Ac-ΔAla-[psi]Imz-5-Me (6)						
Conformer	*φ*	*Ψ*	*E*	Δ*E*	Δ*Eτ/π*	*p* [%]	Conformer	*Φ*	*ψ*	E	Δ*E*	Δ*Eτ*/*π*	*p* [%]
*Gas Phase*							*Gas Phase*						
*β*2	− 179.3	0.2	− 345,807.0531	0.00	0.00	53.21	C5	− 179.8	− 179.9	− 345,806.9738	0.00	0.08	46.58
*α*D	− 54.4	− 148.9	− 345,803.4036	3.65	3.65	0.12	C7	− 55.1	33.0	− 345,803.2080	3.77	3.85	0.08
C5	− 159.8	− 151.9	− 345,802.1372	4.92	4.92	0.01	*β*2	− 159.9	30.2	− 345,800.3697	6.60	6.68	0.00
*α*	− 47.3	− 36.6	− 345,799.5870	7.47	7.47	0.00	*β*	− 48.0	145.5	− 345,799.3845	7.59	7.67	0.00
					**Sum *p***	**53.34**						**Sum *p***	**46.66**
*Chloroform*							*Chloroform*						
*β*2	− 179.9	0.0	− 345,820.1017	0.00	0.00	55.80	C5	− 179.5	− 178.2	− 345,819.9606	0.00	0.14	44.03
*α*D	− 58.8	− 147.5	− 345,815.9950	4.11	4.11	0.06	*β*2	− 155.0	28.4	− 345,815.8766	4.08	4.23	0.05
C5	− 152.6	− 154.2	− 345,815.6358	4.47	4.47	0.03	*α*	− 60.2	− 10.1	− 345,815.5177	4.44	4.58	0.03
*α*	− 53.3	− 30.0	− 345,814.6544	5.45	5.45	0.01	*β*	− 53.2	153.0	− 345,814.6078	5.35	5.49	0.01
					**Sum *p***	**55.89**						**Sum *p***	**44.11**
*Water*							*Water*						
*β*2	− 179.5	0.1	− 345,819.3488	0.00	0.30	34.40	C5	− 178.9	− 174.5	− 345,819.6459	0.00	0.00	56.67
*β*	− 56.7	157.4	− 345,817.3512	2.00	2.29	1.20	*β*	− 55.2	151.7	− 345,817.6764	1.97	1.97	2.07
*α*	− 54.5	− 31.3	− 345,817.2806	2.07	2.38	1.07	*α*	− 56.6	− 25.2	− 345,817.6685	1.98	1.98	2.05
C5	− 144.3	− 155.4	− 345,817.1300	2.22	2.52	0.83	*β*2	− 141.5	24.9	− 345,817.5629	2.08	2.08	1.71
					**Sum *p***	**37.50**						**Sum *p***	**62.50**

The tautomer *π* for this residue should be again considered. In the gas phase, the tautomer *π*, Ac-ΔAla-[psi]Imz-5-Me (**6**) adapts the conformations C5 (*φ*, *ψ* ~ − 179.8°, − 179.9°) and C7 (*φ*, *ψ* ~ − 55.1°, 33.0°), as well as the conformations *β*2 (*φ*, *ψ* ~ − 159.9°, 30.2°) and *β* (*φ*, *ψ* ~ − 48°, 145.5°), and their mirror counterparts.

**Fig. 8:**
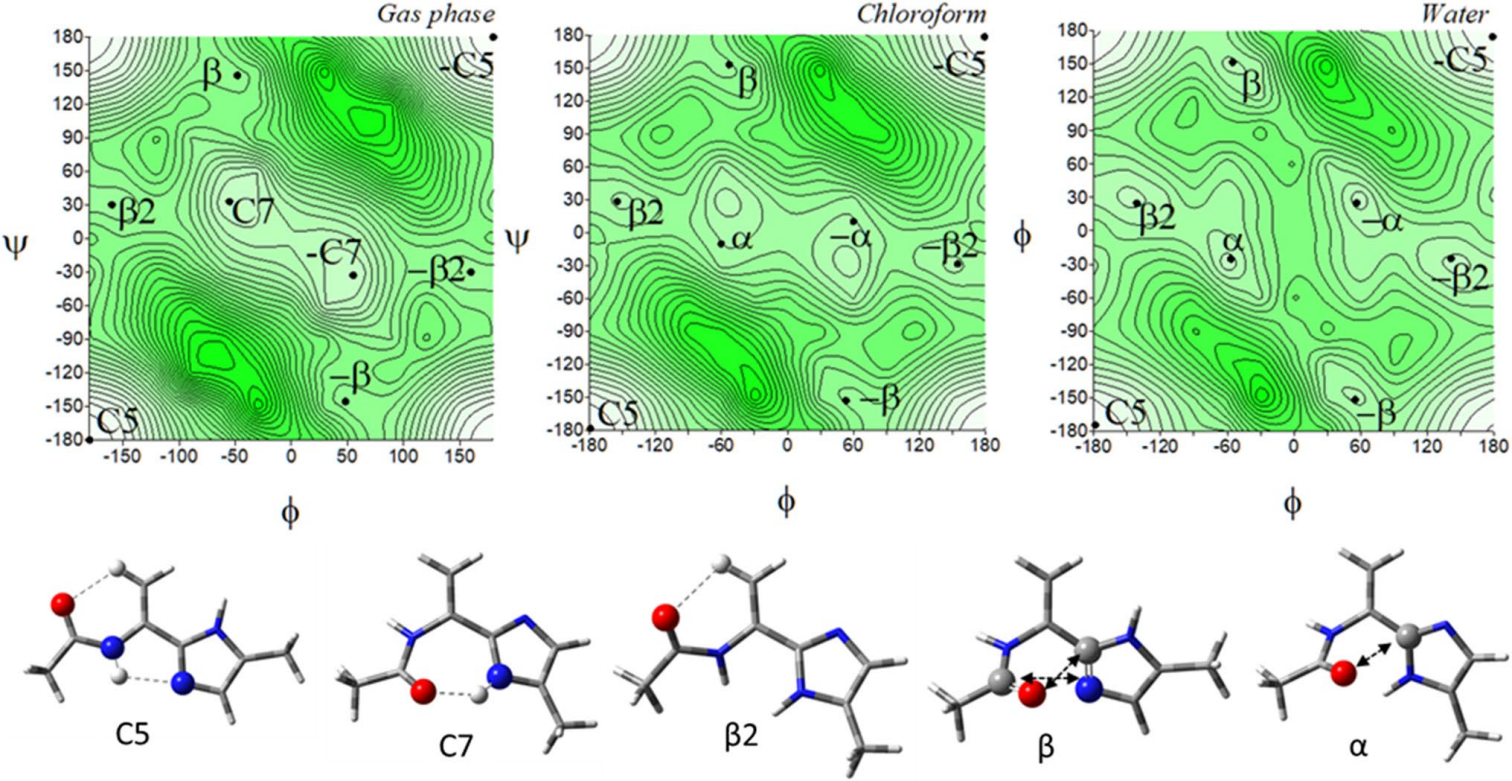
The potential energy surfaces *E* = *f*(*φ*,*ψ*) for Ac-ΔAla-[psi]Imz-5-Me (**6**) calculated at M06-2X/6–31+G(d,p) method in the gas phase, chloroform, and water. Energy contours are plotted every 1 kcal/mol. The darker colour indicates the high in energy regions and the lighter the low in energy regions. Conformations optimised in water with the most important electrostatic interactions (◄⋯►) and hydrogen bonds (⋯) created within the residue

In weakly polar chloroform, the conformation C7 vanishes and the conformation *α* (*φ*, *ψ* ~ − 60.2°, − 10.1°) appears. In terms of internal interactions, created within the studied residue, the conformations of the tautomer *π* correspond to those of the tautomer *τ*. The conformation C5 (**6**) is stabilized in the same way as the conformation *β*2 (**5**), the conformation *α* as the conformation *β*, and the conformation C7 as the conformation *α*D. The torsion angles *φ* of the presented pairs of conformations are almost the same; contrariwise, the torsion angles *ψ* differ by around 180°. The energy difference within the pairs of these conformations is very small. Thus, in the environment, they should occur in equilibrium. Moreover, some of the conformers overlap, for example, *β*2 and C5 (Figs. 7 and 8). Likewise, for the tautomers of the saturated analogue, tautomerization causes a turn of the imidazole ring to create stable intramolecular interactions. As can be seen, the most considerable impact of tautomerisation is the adjustment of the peptide chain conformation. A comparison of the most stable conformations, C5 (**6**) and *β*2 (**5**) shows that the tautomer *π* would create a linear conformation of the main chain and tautomer *τ*—a turn.

Comparing the conformational properties of compounds (**1**) and (**5**), it seems that dehydroamino acid has less conformational freedom, especially in water. The unsaturated bond in the side chain and, as a consequence, the presence of the *π*-electron conjugation between the atoms stabilize the conformations *β*2/C5 (*τ*/*π*) so well that these conformations prevail even in water (91%), in contrast to saturated analogue where the global conformations are *α*R/C7eq.

Shapes of the maps of ΔAla-[psi](Ozl) (Siodłak et al. 2014a), ΔAla-[psi](Tzl) (Staś et al. 2021) and ΔAla and the presented ΔAla-[psi](Imz) are very alike. The same conformations are available in all mimicked environments. Nevertheless, the imidazole tautomer *τ* (**5**) has the strongest tendency toward the conformation *β*2, and its population in water is the highest. It can be also concluded that dehydroamino acids with the heterocycle in the main chain can form a strong intramolecular hydrogen bonds N–H···N^Xzl^, and among the studied azoles the strongest one forms the imidazole residues.

## Imidazolium-dehydroalanine (7)

**Fig. 9:**
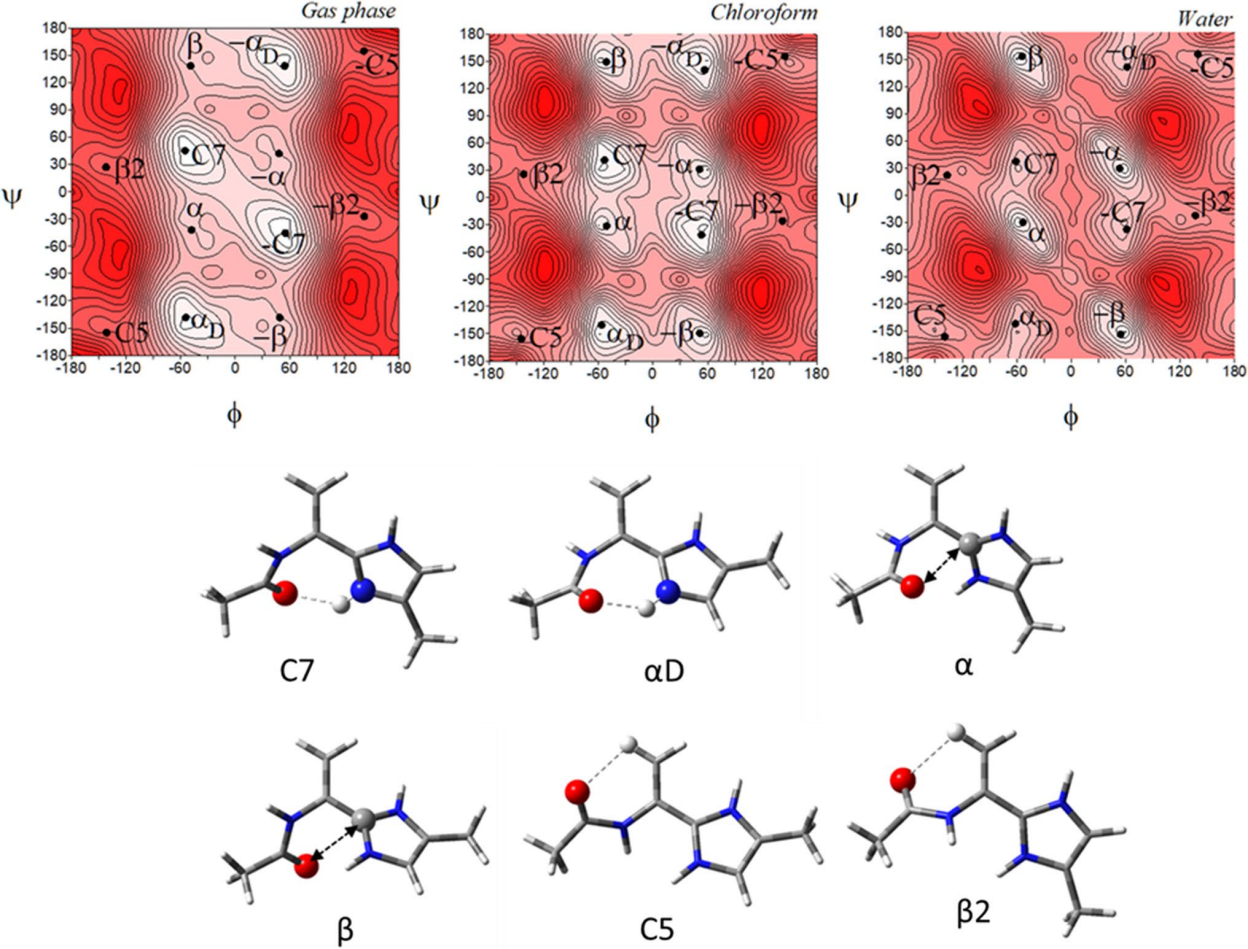
The potential energy surfaces *E* = *f*(*φ*,*ψ*) for Ac-ΔAla-[psi](Imz)^+^-4-Me (**7**) calculated at M06-2X/6–31+G(d,p) method in the gas phase, chloroform, and water. Energy contours are plotted every 1 kcal/mol. The darker colour indicates the high in energy regions and the lighter—low in energy. Below maps are conformations optimised in water with the most important electrostatic interactions (◄⋯►) and hydrogen bonds (⋯) created within the residue

The potential energy surfaces for the imidazolium-dehydroalanine, Ac-ΔAla-[psi](Imz)^+^-4-Me (**7**) are shown in Fig. 9. Regardless of the environment, six conformations are present on maps: C7, *α*D, *α*, *β*, C5, and *β*2 as well as their mirror analogues. In the gas phase, the lowest energy has the conformations C7 (*φ*, *ψ* = − 55.2°, 45.3°) and *α*D (*φ*, *ψ* = − 54.4°, − 138.5°), populated almost 98% (Table 5) and stabilised by the N–H···O hydrogen bonds involved imidazolium N–H group. As the polarity of the simulated environment increases, the tendency towards the conformations *β* and *α* is observed, stabilized by dipole interactions, and they prevail finally in the water. These conformations are mainly located in the middle of the map, with the average value of the torsion angle *φ* about − 55° (and 55°, respectively). The same feature is present in the saturated analogue. Two other conformations, C5 and *β*2 have relatively highest or higher energy and are scarcely populated. They are stabilised by the C^*β*^-H···O hydrogen bonds. The proximity of the N-terminal amide and C-terminal imidazolium N–H groups indicates disadvantageous H···H repulsion and makes impossible the N–H···N hydrogen bond, which stabilizes analogous conformations for neutral forms (**5**) and (**6**).

**Table 5:** Selected torsion angles (°) of local minima for Ac-ΔAla-[psi](Imz)^+^-4-Me (**8**), optimized at M06-2X/6–311++G(d,p) method, their relative energies (Δ*E*) and the relative energy difference between tautomers (Δ*Eτ/π*) in kcal/mol, and population (*p*)

Ac-ΔAla-[psi](Imz)^+^-4-Me (7)					
Conformer	*φ*	*ψ*	E [a.u.]	ΔE	*p* [%]
*Gas Phase*					
C7	− 55.2	45.3	− 551.2702	0.00	50.67
*α*D	− 54.4	− 138.5	− 551.2701	0.05	46.98
*α*	− 48.0	− 42.1	− 551.2668	2.22	1.22
*β*	− 49.1	138.6	− 551.2667	2.26	1.13
C5	− 141.6	− 155.0	− 551.2579	7.95	0.00
*β*2	− 142.2	27.1	− 551.2578	8.05	0.00
*Chloroform*					
C7	− 53.4	41.2	− 551.3481	0.00	45.29
*α*D	− 56.6	− 140.5	− 551.3480	0.29	27.75
*β*	− 51.3	149.4	− 551.3477	0.66	14.86
*α*	− 51.1	− 31.3	− 551.3476	0.85	10.95
C5	− 144.8	− 155.6	− 551.3443	2.58	0.60
*β*2	− 142.3	25.8	− 551.3442	2.62	0.56
*Water*					
*β*	− 54.1	153.4	− 551.3603	0.00	43.01
*α*	− 53.2	− 29.6	− 551.3602	0.15	33.46
C5	− 139.4	− 156.0	− 551.3585	0.99	8.20
*β*2	− 136.8	22.2	− 551.3585	1.00	8.06
*α*D	− 61.5	− 141.7	− 551.3579	1.39	4.19
C7	− 61.2	37.6	− 551.3578	1.57	3.08

Surprisingly, the imidazolium-dehydroalanine residue (**7**) has more available conformations than the saturated imidazolium-alanine (**3**). It seems it is due to the presence of the *π*-electron conjugation, which stabilises the conformations C5 and *β*2 in the compound (**7**). However, the low-energy conformation is alike in the studied environments. The global conformations for molecule (**3**) are C7 and *α*D in the gas phase and *β* and C7 in chloroform and water. For the compound (**7**): C7 and *α*D in the gas phase and chloroform and *α* and *β* in water. The preferable torsion angle *φ*, in molecule (**3**) is about − 75° and in molecule 7, it is about − 55°. The angle diminishes because of the lack of steric hindrance in the side chain. Due to this, also the torsion angle *ψ* is very flexible.

## Imidazolide-dehydroalanine (8)

**Fig. 10:**
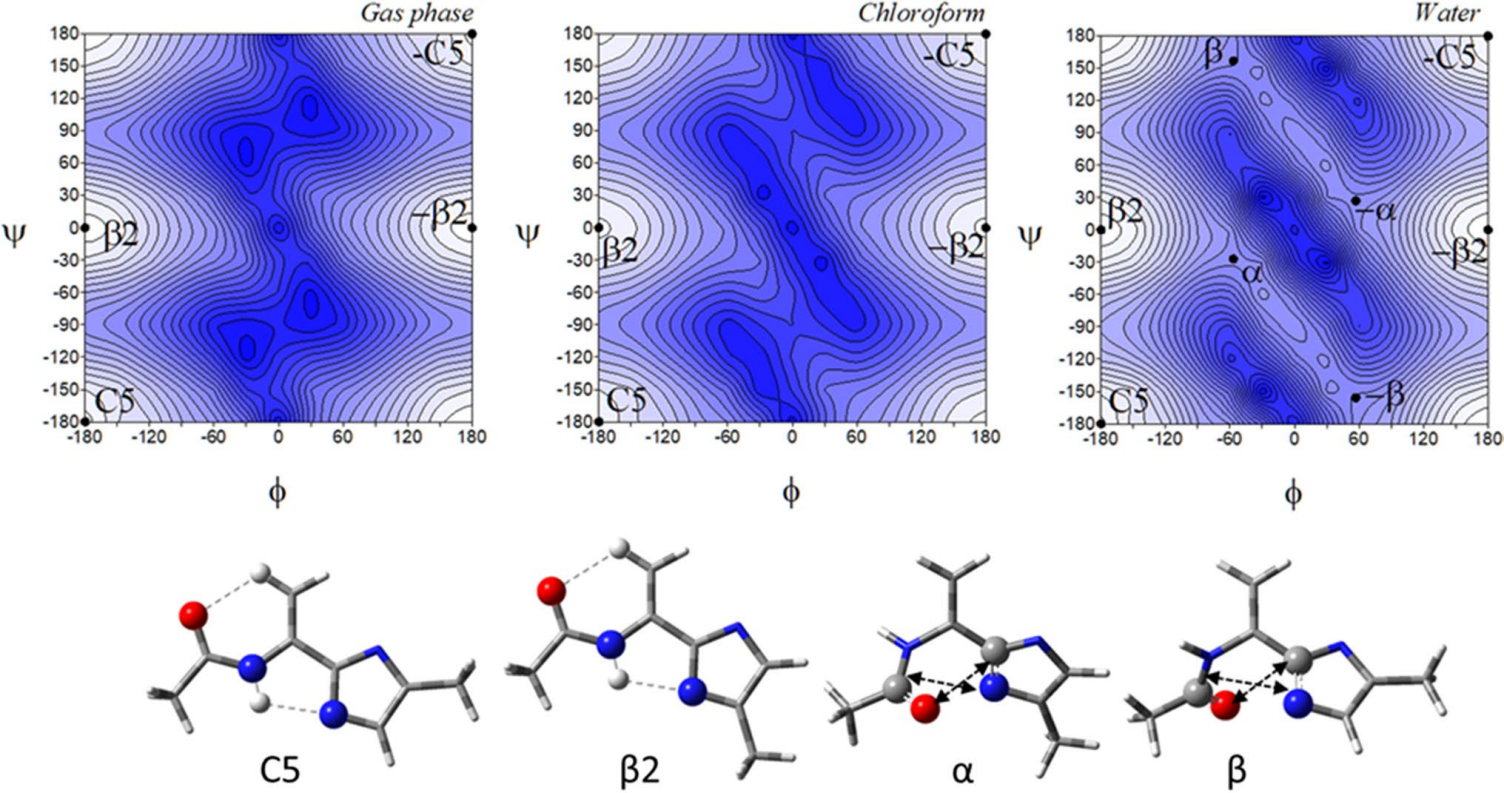
The potential energy surfaces *E* = *f*(*φ*,*ψ*) for Ac–ΔAla-[psi](Imz)^−^-4-Me (**8**) calculated at M06-2X/6–31+G(d,p) method in the gas phase, chloroform, and water. Energy contours are plotted every 1 kcal/mol. The darker colour indicates the high in energy regions and the lighter—low in energy. Below maps are conformations optimised in water with the most important electrostatic interactions (◄⋯►) and hydrogen bonds (⋯) created within the residue

Figure 10 presents the conformational maps of the negatively charged analogue of imidazole-dehydroalanine, Ac-ΔAla-[psi](Imz)^−^-4-Me (**8**). For this molecule, four different conformations and their mirror counterparts are found: *β*2, C5, *β*, and *α*. The conformations *α* (*φ*, *ψ* = − 57.0°, − 27.1°) and *β* (*φ*, *ψ* = − 57.9°, 156.8°) are predicted only in the water environment. The conformations *β*2 (*φ*, *ψ* = -180.0°, 0.0°) and C5 (*φ*, *ψ* = − 180.0°, − 180.0°) are of the greatest importance. As is shown in Table 6, the conformation C5 has a slight energy advantage over the conformation *β*2, which is not clearly shown by the difference in the energy but is expressed by the population percentage. The presence of these two conformations is caused by the fact that both nitrogen atoms of the imidazole can create the same hydrogen bonds within the residue: N–H···N^Imz^ and C^*β*^-H···O. There is a great similarity between this conformational profile and the imidazole-alanine anion (**4**). Nevertheless, for the imidazole-dehydroalanine (**8**), the *π*-electron conjugation stabilization force is gained by the alignments in the plane of all required atoms. This shifts the conformations towards the edges of the maps. Moreover, the geometry of conformations is rigid. It does not change even in water. Both imidazolide-alanine (**4**) and imidazolide-dehydroalanine (**8**) anions incline the conformations *β*2 and C5. The saturation of the side chain and the environment slightly influence these propensities.

**Table 6:** Selected torsion angles (°) of local minima for Ac-ΔAla-[psi](Imz)^−^-4-Me (**8**), optimized at M06-2X/6–311++G(d,p) method, their relative energies (Δ*E*) and the relative energy difference between tautomers (Δ*Eτ*/*π*) in kcal/mol, and population (*p*)

Ac-ΔAla-[psi](Imz)^−^-4-Me (8)					
Conformer	*φ*	*Ψ*	E [a.u.]	ΔE	*p* [%]
*Gas Phase*					
C5	− 180.0	− 180.0	− 550.3435	0.00	60.54
*β*2	− 180.0	0.0	− 550.3432	0.25	39.46
*Chloroform*					
C5	− 180.0	− 180.0	− 550.4177	0.00	55.59
*β*2	− 180.0	0.0	− 550.4175	0.13	44.41
*Water*					
C5	− 180.0	− 180.0	− 550.4346	0.00	65.35
*β*2	− 180.0	0.0	− 550.4344	0.38	34.39
*α*	− 57.0	− 27.1	− 550.4290	3.70	0.13
*β*	− 57.9	156.8	− 550.4290	3.72	0.13

### Conclusions

The conformational properties of the imidazole-alanine and imidazole-dehydroalanine, as the representatives of non-standard amino acid residues with the C-terminal peptide group replaced by imidazole, were determined. For imidazole, both neutral forms (tautomers *τ* and *π*) and charged forms (protonated and deprotonated) were considered.

Regardless of the protonation state of imidazole, the studied amino acid residues have a pair of low-energy conformations, for which the position of imidazole ring changes by about half a turn (~ 180°). This can be explained by the presence of hydrogen bonds created within the studied residue, in which the imidazole acts as the hydrogen bond donor or acceptor. With exception of the protonated imidazolium-dehydroalanine (**7**), the conformational properties of the studied molecular motifs (**1–6**, and **8**) basically do not depend on the polarity of the environment. This proves the extraordinary stability of the internal hydrogen bonds created by the imidazole, which must compete in a polar environment with growing intermolecular interactions, but still, they are maintained within the studied residues and stabilises the lowest in energy conformations. As these lowest energy conformations, both for neutral tautomers as well as protonated and deprotonated forms, differ only in rotation of the imidazole ring and the same set of hydrogen bonds is formed, this indicates that the choice of the conformation does not depend on the stabilising forces created within the studied residues, but rather on remote interactions, within the bigger molecule or due to molecular association. This creates an interesting conformational switch, in which the changes in the geometry of the amino acid residue can result in changes in the 3-dimensional structure of the peptide.

The described imidazole-amino acids can be applied, potentially, to design pH-sensitive peptides for targeting acidic tissues (Furukawa et al. 2017; Weerakkody et al. 2013), or to use cell surface acidity as a feature that can be exploited using pH-sensitive peptide folding to target agents to diseased cell surfaces or cytoplasms (Reshetnyak et al. 2020).

To obtain the presented preliminary results, the DFT method was applied, as the theoretical calculations give a relatively brief, but complete image of these structural properties. It is also a good starting point for experimental studies, which should verify the presented results.

## Supplementary Information

Below is the link to the electronic supplementary material.

Supplementary file1 (PDF 876 KB)
