# Supplementary material for: Imidazole-amino acids. Conformational switch under tautomer and pH change
Source: Amino Acids. 2022 Nov 2;55(1):33–49. doi: 10.1007/s00726-022-03201-0 (PMC9877100; doi:10.1007/s00726-022-03201-0)
Supplement: Supplementary file 1 — Supplementary file1 (PDF 876 KB) [file 726_2022_3201_MOESM1_ESM.pdf]

SUPPORTING INFORMATION for

Imidazole-amino acids. Conformational switch under tautomer and pH change

Monika Staś*, Piotr Najgebauer, Dawid Siodłak

Faculty of Chemistry, University of Opole, Opole 45-052, Poland

e-mail: mstas@uni.opole.pl

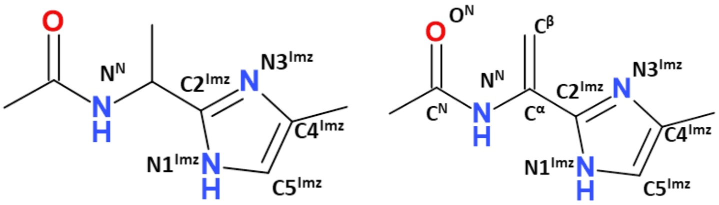

Fig. S1 Schematic formula of imidazole-alanine and imidazole-dehydroalanine with atoms notation

Table S1 Structural parameters for the internal X–H···A (X = N, C; A = O, N) hydrogen bond; H···A ≤ 2.7 Å and X-H···A > 90º according to Ref. (Vargas et al. 2002), at the M06-2X/6-311++G(d,p) conformers for the molecules **1, 2, 3** and **4** in water

Conformer Code	**Ac-L-Ala–[psi]Imz-4-Me τ (1)**				**Ac-L-Ala–[psi]Imz-5-Me π (2)**					**Ac-L-Ala–[psi](Imz)$^+$-4-Me (3)**						**Ac-L-Ala–[psi](Imz)$^-$-4-Me (4)**			
	**αRτ**	**β2**	**αLτ**	**α'**	**C7eq**	**C7ax**	**C5**	**β2π**	**αLπ**	**αRτ**	**C7eq**	**αLπ**	**C7ax**	**αLτ**	**αDτ**	**C5**	**β2**	**αLπ**	**αDτ**
**N^N-H···X**							**X=N3^{Imz}**									**X=N3^{Imz}**	**X=N1^{Imz}**		
r H··· **X**							2.32									2.32	2.35		
r N··· **X**							2.76									2.77	2.79		
N-H··· **X**							104.61									105.9	105.5		
∠H···X-C2^{Imz}							80.60									80.6	79.7		
**O···H-NY^{Iml}**	**Y=3**		**Y=3**		**Y=1**	**Y=1**				**Y=3**	**Y=1**		**Y=1**	**Y=3**					
r H··· **Y**	2.16		2.06		2.16	2.06				2.08	2.08		1.99	1.98					
r N··· **Y**	2.92		2.84		2.92	2.84				2.84	2.85		2.78	2.77					
∠O···H-NY^{Imz}	130.5		132.0		130.51	131.37				130.3	15.8		132.1	132.1					
∠C=O···H^l	107.8		113.3		107.43	114.07				111.8	11.4		116.2	116.5					
**C^α-H··· O^N**																			
r H···O	2.54	2.33		2.52	2.54		2.57	2.50											
r C···O	2.80	2.76		2.79	2.80		2.79	2.79											
∠C-H···O	92.1	104.5		92.7	92.25		89.52	93.79											
∠C=O···H	79.4	80.6		80.3	79.43		79.04	80.57											
**C^β-H···O^N**																			
r H···O			2.52		-	2.53	2.72		2.65			2.65	2.52		2.64			2.70	2.70
r C···O			3.08		-	3.09	3.26		3.19			3.18	2.78		3.17			3.23	3.23
∠C-H···O			110.8		-	110.66	110.02		110.03			109.2	132.1		109.3			109.7	109.6
∠C=O···H			97.5		-	97.52	88.10		92.97			92.6	116.2		92.7			91.8	91.7

Table S2 Structural parameters for the internal X–H···A (X = N, C; A = O, N) hydrogen bond; H···A ≤ 2.7 Å and X-H···A > 90 ° according to Ref. (Vargas et al. 2002), at the M06-2X/6-311++G(d,p) conformers for the molecules **5, 6, 7** and **8** in water

Conformer Code	**Ac–ΔAla–[psi]Imz-4-Me τ (5)**		**Ac–ΔAla–[psi]Imz-5-Me π (6)**		**Ac–ΔAla–[psi](Imz)$^{+}$-4-Me (7)**				**Ac–ΔAla–[psi](Imz)$^{-}$-4-Me (8)**	
	**β2**	**C5**	**C5**	**β2**	**C5**	**β2**	**αD**	**C7**	**β2**	**C5**
**N^{N}-H···X**	**X=N1^{Imz}**		**X=N3^{Imz}**	**X=N1^{Imz}**				**X=N1^{Imz}**	**X=N1^{Imz}**	**X=N3^{Imz}**
r H··· **X**	2.21		2.22	2.67				1.92	2.14	2.14
r N··· **X**	2.72		2.73	2.84				2.73	2.69	2.69
∠N-H··· **X**	109.6		109.3	88.98				134.6	111.8	111.6
∠ H···X-C2^{Imz}	81.1		80.73	71.78				112.0	82.8	82.8
**O···H-NY^{Imz}**							**Y=3**			
r H··· **Y**							1.91			
r N··· **Y**							2.73			
∠O···H-NY^{Imz}							134.7			
∠C=O···H^{I}							112.2			
**C^{β}-H···O^{N}**										
r H···O	2.27	2.50		2.53	2.54	2.57			2.29	2.29
r C···O	2.89	2.94		2.95	2.94	2.95			2.91	2.91
∠C-H···O	114.8	103.3		101.88	100.9	99.5			114.9	114.8
∠C=O···H	104.9	95.3		94.01	94.5	93.3			103.9	104.1
**N^{N}···H-NY^{Imz}**				**Y=1**						
r H··· **Y**				2.66						
r N··· **Y**				2.84						
∠N···H-NY^{Imz}				89.89						
∠ C^{α}-N···H^{I}				80.34						

Table S3 Structural parameters for the internal C=O ►···◄ C=O interactions; C···O < 3.6Å according to Ref. (Allen et al. 1998), at the M06-2X/6-311++G(d,p) conformers for the molecules **1,2,3 and 4** in water

	**Ac-L-Ala–[psi] Imz-4-Me, τ (1)**		**Ac-L-Ala–[psi] Imz-5–Me, π (2)**				**Ac-L-Ala–[psi] (Imz)$^{+}$-4-Me (3)**				**Ac-L-Ala–[psi] (Imz)$^{-}$-4-Me (4)**	
**Conformer Code**	**β**	**αDτ**	**C7eq**	**αRπ**	**C7ax**	**αLp**	**β**	**αRπ**	**αLπ**	**αDτ**	**αDτ**	**αLπ**
	**Y=3**	**Y=3**	**Y=1**	**Y=1**	**Y=3**	**Y=3**	**Y=3**	**Y=1**	**Y=1**	**Y=3**	**Y=1**	**Y=3**
O^{N}···C2^{Imz}	3.03	2.95	3.26	3.01	3.26	2.93	2.93	2.91	2.82	2.82	3.00	3.00
C^{N}···NY^{Imz}	-	-	3.25	3.32	3.25	3.26	-	-	-	-	3.44	3.50
C^{N}···C2^{Imz}	3.05	3.02	3.18	3.04	3.18	3.02	3.00	2.99	2.95	2.96	3.05	3.06
O^{N}··· NY^{Imz}	-	-	2.92	3.43	2.92	3.28	-	-	-	-	3.52	3.59
∠(C=O)^{N}··· C2^{Imz}	79.5	81.4	75.45	79.93	75.45	81.84	81.3	81.8	83.9	83.9	80.5	80.6
∠(C2=NY)^{Imz}···C^{N}	66.8	68.8	75.07	66.61	75.07	67.62	64.6	63.4	63.6	64.6	62.1	60.0
∠NY^{Imz}···(C=O)^{N}	85.3	80.0	63.89	84.55	63.89	79.90	81.6	81.3	76.8	76.4	83.7	84.2
∠O^{N}···(C2-NY)^{Imz}	96.3	90.6	63.73	96.1	63.73	92.10	97.5	99.0	96.1	94.6	101.6	104.9

Table S4 Structural parameters for the internal C=O ►···◄ C=O interactions; C···O < 3.6Å according to Ref. (Allen et al. 1998), at the M06-2X/6-311++G(d,p) conformers for the molecules **1,2,3 and 4** in water

	**Ac–ΔAla–[psi]Imz-4–Me τ(5)**		**Ac–ΔAla–[psi]Imz-5-Me π(6)**		**Ac–ΔAla–[psi](Imz)$^{+}$ -4-Me (7)**		**Ac–ΔAla–[psi](Imz)$^{-}$-4-Me (8)**	
**Conformer Code**	**α**	**β**	**β**	**α**	**α**	**β**	**β**	**A**
	**Y=1**	**Y=3**	**Y=3**	**Y=1**	**Y=1**	**Y=3**	**Y=3**	**Y=1**
O^{N}···C2^{Imz}	2.95	2.96	2.87	3.04	3.04	3.05	2.87	2.86
C^{N}···NY^{Imz}	3.28	-	-	3.25	3.25	3.24	-	-
C^{N}···C2^{Imz}	3.03	3.05	3.00	3.08	3.08	3.08	3.00	3.00
O^{N}··· NY^{Imz}	3.22	-	-	3.24	3.24	3.25	-	-
∠(C=O)^{N}··· C2^{Imz}	82.0	82.5	84.1	80.3	80.3	79.9	84.1	84.3
∠(C2=NY)^{Imz}···C^{N}	67.6	71.6	68.5	70.9	70.9	71.2	68.5	68.3
∠NY^{Imz}···(C=O)^{N}	76.2	75.0	85.3	78.9	78.9	79.6	85.3	71.9
∠O^{N}···(C2=NY)^{Imz}	89.3	83.5	86.2	86.3	86.3	86.3	86.2	86

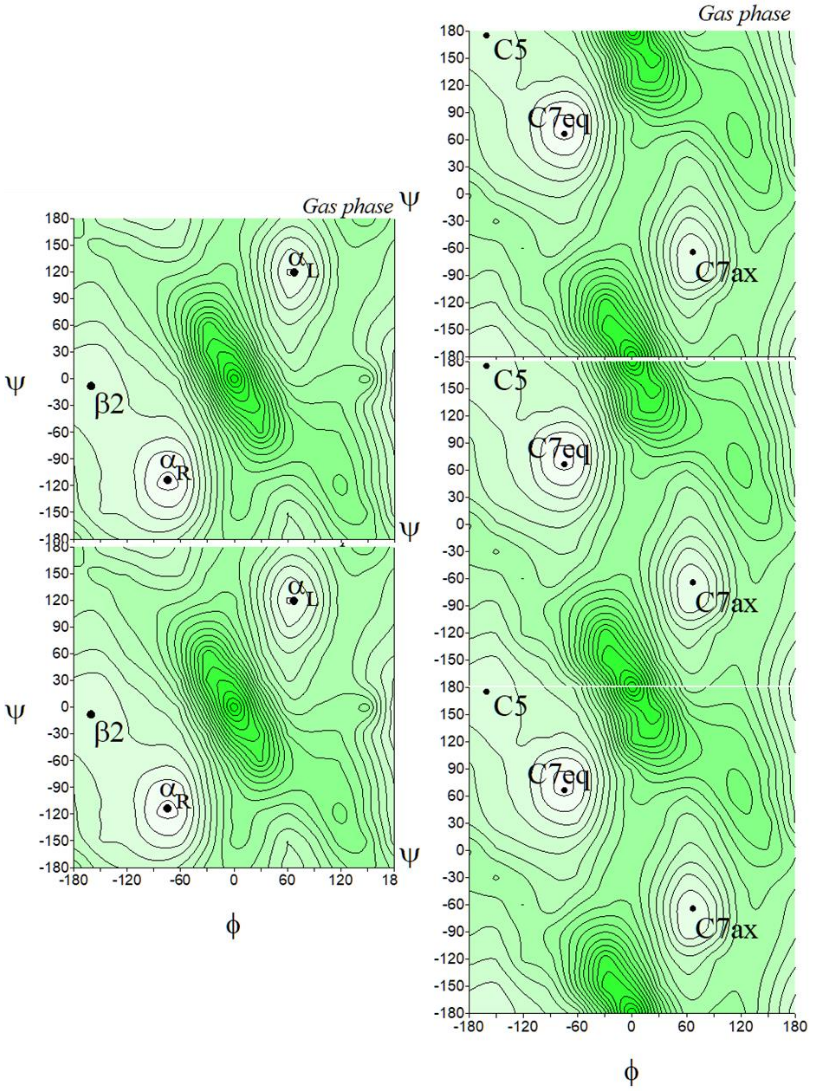

Fig. S2. The potential energy surfaces E=f(φ,ψ) of Ac-L-Ala-[psi]Imz-4-Me (**1**, left) and Ac-L-Ala-[psi]Imz-5-Me (**2**, right) calculated at M06-2X/6-311++G(d,p) method in the gas phase. Energy contours are plotted every 1 kcal/mol. The darker colour indicates the high in energy regions and the lighter - low in energy regions.

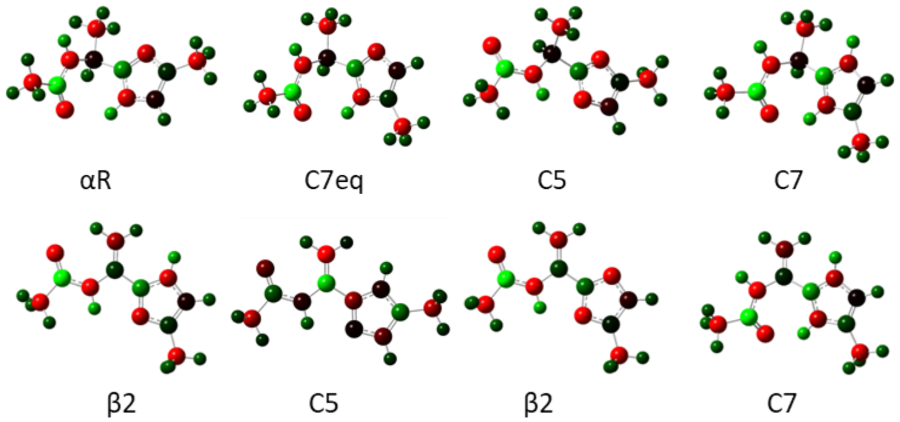

Fig. S3 Visual representation of NBO charges for the studied compounds in lowest conformation calculated in gas phase at M06-2X/6-311++G(d,p) level of theory. The colures range from green – (positive charge) to red (negative charge)

Table S5 The NBO charges of selected atoms (according to drawings) for the studied compounds in all conformations calculated in gas phase at M06-2X/6-311++G(d,p) level of theory

	**O^{N}**	**C^{N}**	**N^{N}**	**H^{N}**	**N1^{Imz}**	**H1^{Imz}**	**C2^{Imz}**	**N3^{Imz}**	**H3^{Imz}**	**C4^{Imz}**	**C5^{Imz}**
**Ac-L-Ala-[psi]Imz-4-Me (1)**											
**αRτ**	-0.69	0.71	-0.65	0.40	-0.56	-	0.38	-0.58	0.45	-0.10	0.08
**αLτ**	-0.69	0.71	-0.65	0.40	-0.53	-	0.37	-0.56	0.45	-0.09	0.10
**β2**	-0.66	0.71	-0.67	0.43	-0.55	-	0.40	-0.56	0.42	-0.09	0.11
**Ac-L-Ala-[psi]Imz-5-Me (2)**											
**C7eq**	-0.69	0.71	-0.65	0.40	-0.57	0.45	0.37	-0.52	-	-0.09	0.11
**C7ax**	-0.69	0.71	-0.65	0.40	-0.58	0.45	0.37	-0.51	-	-0.09	0.11
**C5**	-0.66	0.70	-0.67	0.43	-0.57	0.41	0.40	-0.54	-	-0.07	0.11
**Ac-L-Ala-[psi](Imz)$^{+}$-4-Me (3)**											
**C7eq**	-0.68	0.73	-0.66	0.41	-0.52	0.49	0.49	-0.50	0.45	-0.04	0.18
**αRτ**	-0.68	0.73	-0.66	0.41	-0.51	0.45	0.48	-0.50	0.49	-0.03	0.16
**C7ax**	-0.69	0.73	-0.66	0.41	-0.52	0.49	0.48	-0.50	0.45	-0.04	0.17
**αDτ**	-0.69	0.73	-0.66	0.42	-0.51	0.44	0.48	-0.51	0.50	-0.03	0.16
**αLπ**	-0.62	0.71	-0.69	0.42	-0.51	0.49	0.52	-0.50	0.45	-0.04	0.17
**αDτ**	-0.62	0.71	-0.69	0.42	-0.51	0.45	0.52	-0.50	0.45	-0.03	0.17
**β**	-0.62	0.71	-0.70	0.41	-0.51	0.45	0.52	-0.51	0.45	-0.09	0.17
**αRπ**	-0.62	0.71	-0.70	0.41	-0.52	0.45	0.52	-0.50	0.45	-0.09	0.17
**Ac-L-Ala-[psi](Imz)$^{-}$-4-Me (4)**											
**C5**	-0.70	0.69	-0.66	0.44	-0.62	-	0.30	-0.65	-	-0.14	0.04
**β2**	-0.70	0.69	-0.65	0.44	-0.67	-	0.30	-0.60	-	-0.14	0.04
**Ac-ΔAla-[psi]Imz-4-Me (5)**											
**β2**	-0.63	0.71	-0.66	0.43	-0.59	-	0.36	-0.54	0.42	-0.07	0.11
**β**	-0.67	0.72	-0.65	0.40	-0.51	-	0.34	-0.55	0.46	-0.08	0.11
**C5**	-0.61	0.70	-0.67	0.40	-0.50	-	0.35	-0.56	0.42	-0.09	0.11
**α**	-0.61	0.71	-0.67	0.40	-0.51	-	0.36	-0.54	0.42	-0.08	0.11
**Ac-ΔAla-[psi]Imz-5-Me (6)**											
**C5**	-0.63	0.71	-0.65	0.43	-0.55	0.42	0.36	-0.53	-	-0.07	0.12
**C7**	-0.67	0.72	-0.65	0.40	-0.56	0.46	0.33	-0.50	-	-0.08	0.12
**β2**	-0.61	0.70	-0.67	0.39	-0.57	0.41	0.34	-0.48	-	-0.07	0.11
**β**	-0.61	0.71	-0.68	0.40	-0.55	0.42	0.35	-0.49	-	-0.07	0.12
**Ac-ΔAla-[psi](Imz)$^{-}$-4-Me (8)**											
**β2**	-0.67	0.69	-0.65	0.44	-0.64	-	0.26	-0.57	-	-0.12	0.05
**C5**	-0.68	0.69	-0.65	0.44	-0.59	-	0.26	-0.68	-	-0.13	0.06
**Ac-ΔAla-[psi](Imz)$^{+}$-4-Me (7)**											
**C7**	-0.67	0.74	-0.66	0.42	-0.51	0.49	0.44	-0.50	0.45	-0.03	0.18
**αD**	-0.67	0.74	-0.66	0.42	-0.51	0.45	0.45	-0.49	0.50	-0.03	0.17
**A**	-0.60	0.71	-0.68	0.42	-0.50	0.45	0.48	-0.50	0.45	-0.03	0.18
**β**	-0.60	0.72	-0.69	0.42	-0.50	0.45	0.48	-0.49	0.45	-0.03	0.17
**C5**	-0.57	0.72	-0.71	0.41	-0.51	0.45	0.46	-0.49	0.46	-0.03	0.18
**β2**	-0.57	0.72	-0.72	0.41	-0.50	0.45	0.46	-0.50	0.45	0.03	0.17
**Ac-ΔAla-[psi](Imz)$^{-}$-4-Me (8)**											
**β2**	-0.67	0.69	-0.65	0.44	-0.64	-	0.26	-0.57	-	-0.12	0.05
**C5**	-0.68	0.69	-0.65	0.44	-0.59	-	0.26	-0.68	-	-0.13	0.06

References

Allen FH, Baalham CA, Lommerse JPM, Raithby PR (1998) Carbonyl-Carbonyl Interactions can be Competitive with Hydrogen Bonds. Acta Crystallographica Section B: Structural Science 54 (3):320-329.

Vargas R, Garza J, Hay BP, Dixon DA (2002) Conformational study of the alanine dipeptide at the MP2 and DFT levels. Journal of Physical Chemistry A 106 (13):3213-3218.
